# The interplay between temperature, *Trypanosoma cruzi* parasite load, and nutrition: Their effects on the development and life-cycle of the Chagas disease vector *Rhodnius prolixus*

**DOI:** 10.1371/journal.pntd.0011937

**Published:** 2024-02-02

**Authors:** Henri Loshouarn, Alessandra A. Guarneri

**Affiliations:** Vector Behavior and Pathogen Interaction Group, Instituto René Rachou, Fundação Oswaldo Cruz-FIOCRUZ, Belo Horizonte, Brazil; Tulane University School of Public Health and Tropical Medicine, UNITED STATES

## Abstract

Chagas disease, caused by the protozoan parasite *Trypanosoma cruzi* transmitted by blood-sucking insects of the subfamily Triatominae, is a major neglected tropical disease affecting 6 to 7 million of people worldwide. *Rhodnius prolixus*, one of the most important vectors of Chagas disease in Latin America, is known to be highly sensitive to environmental factors, including temperature. This study aimed to investigate the effects of different temperatures on *R*. *prolixus* development and life-cycle, its relationship with *T*. *cruzi*, and to gather information about the nutritional habits and energy consumption of *R*. *prolixus*. We exposed uninfected and infected *R*. *prolixus* to four different temperatures ranging from 24°C to 30°C, and monitored their survival, developmental rate, body and blood meal masses, urine production, and the temporal dynamics of parasite concentration in the excreted urine of the triatomines over the course of their development. Our results demonstrate that temperature significantly impacts *R*. *prolixus* development, life-cycle and their relationship with *T*. *cruzi*, as *R*. *prolixus* exposed to higher temperatures had a shorter developmental time and a higher mortality rate compared to those exposed to lower temperatures, as well as a lower ability to retain weight between blood meals. Infection also decreased the capacity of the triatomines to retain weight gained by blood-feeding to the next developmental stage, and this effect was proportional to parasite concentration in excreted urine. We also showed that *T*. *cruzi* multiplication varied depending on temperature, with the lowest temperature having the lowest parasite load. Our findings provide important insights into the potential impact of climate change on the epidemiology of Chagas disease, and can contribute to efforts to model the future distribution of this disease. Our study also raises new questions, highlighting the need for further research in order to understand the complex interactions between temperature, vector biology, and parasite transmission.

## Author summary

Chagas disease, caused by the parasite *Trypanosoma cruzi* and transmitted by blood-sucking kissing bugs, is a major neglected tropical disease affecting millions of people worldwide. The insect vector *Rhodnius prolixus* is highly sensitive to environmental factors, including temperature. In this study, we investigated the effects of different temperatures on *R*. *prolixus* and its interaction with *T*. *cruzi*, as well as the nutritional habits and energy consumption of the vector. We exposed uninfected and infected insects to various temperatures, and monitored their survival, development, body and blood meal weights, urine production, and *T*. *cruzi* infection over their lifespan. Our results revealed that temperature significantly influenced *R*. *prolixus* development, life-cycle, and its interaction with *T*. *cruzi*. Insects exposed to higher temperatures had shorter development time, increased mortality, and reduced ability to maintain weight. Infection further compromised the ability of the triatomines to retain weight gained by blood-feeding, and this effect correlated with parasite load. Additionally, *T*. *cruzi* multiplication varied with temperature, with lower temperatures associated with lower parasite concentration. Our findings have important implications for understanding the impact of climate change on Chagas disease epidemiology and contribute to modeling its future spread.

## Introduction

*Rhodnius prolixus* is a hematophagous triatomine insect playing a pivotal role as one of the principal vector species in the domestic transmission of Chagas disease, also known as American trypanosomiasis, primarily within Latin American regions where this parasitic infection is endemic and represents a significant public health challenge [1]. The causative agent of this disease, the protozoan *Trypanosoma cruzi*, is an obligatory parasite infecting both mammals and triatomines, which can function as host reservoirs and/or vectors of the parasite. *Trypanosoma cruzi* transmission commonly occurs through contact with the insect vector, emphasizing the importance of understanding the ecological and biological factors influencing *R*. *prolixus* and its relationship with *T*. *cruzi* [2].

Triatomines acquire *T*. *cruzi* while feeding on an infected mammal, as every instar of these insects feed on blood. Parasite development in the insect is restricted to the intestinal tract, with the rectal ampulla serving as the principal site for the production of infective trypanosome forms [3]. Notably, with regard to chronic infection, very little is known about the temporal population dynamics of the parasite in the rectal ampulla of kissing bugs [4–7]. Chronic infection by *T*. *cruzi* can impact vector fitness and development in several ways, as the parasite exploits vector metabolism, while the presence of the parasite in the triatomine intestine triggers immune responses and alter the composition of the intestinal microbiota [8–12]. Such costs can lead to increases in the duration of the intermolt period of the triatomines [13,14], reduced fecundity, and increased mortality rates [4,15–17]. The extent of the symptoms caused by the presence of the parasite depends on both abiotic environmental and biotic factors, such as the physiological state of the insect, temperature, parasite strain, and/or co-infection with other micro-organisms, such as *Trypanosoma rangeli*, another protozoan parasite of triatomines [14,17–21].

In particular, the nutritional state of triatomines is known to influence the course of *T*. *cruzi* infection of the vector, and its potential adverse effects on insect survival. Starving the vector for a 90-day period is enough to kill 99.5% of the parasites in the rectal ampulla of fifth instar nymphs [3], which may ultimately result in the loss of infection [22]. Additionally, higher temperature-dependent mortality rates have been observed in infected insects 30 days post-blood-feeding, and the survival of infected insects is more heavily impacted by fasting than that of uninfected insects [14,17,22]. These observations suggest that nutritional factors contribute to the higher mortality previously reported in infected insects. Indeed, there is increasing evidence that infection impacts the resource allocation and/or availability of the vector, as triatomines infected by *T*. *cruzi* tend to have lower fertility [16,19].

Triatomines, being ectotherms, are particularly susceptible to the effects of environmental temperature as an abiotic factor, as it influences their physiology, behavior, development, and metabolic rates [23,24]. In addition, as triatomines feed on warm-blooded vertebrate hosts, these insects are also affected by the temperature of the vertebrate blood that they ingest and have undergone adaptative changes to mitigate the risk of heat shock associated with the rapid intake of relatively large volumes of the warm blood of their hosts with otherwise little to no physiological ability to regulate their own body temperature [25]. Consequently, the potential effects of a rise in ambient temperature on their physiology, behavior, and life-history traits remain uncertain, despite being established as the most significant climate change factor affecting insect species [26–28]. These questions are particularly relevant given the fact that the distribution of triatomines and their shelter choice have been shown to be tightly linked to temperature [29–31]. Furthermore, the development of *T*. *cruzi* infection within the intestine of the insect is known to be affected by temperature [3,4,6,14,17], and the multiplication rate of the parasite in culture media is also known to be temperature-dependent [14].

The short-and long-term responses of host-pathogen systems to increasing ambient temperatures both within and over generations is likely to be highly multifactorial, involving intricate trade-offs between nutrition, growth and reproduction of both the pathogens and their hosts/vectors [32,33], potentially leading to notable shifts in the epidemiology and transmission of vector-borne infectious diseases. Anticipated changes include future shifts in the geographical locations of the ecological niches of the parasites/hosts/vectors, potentially giving rise to new areas of disease risk [34,35]. Concurrently, alterations in the population dynamics of vectors in current endemic areas are expected. This might manifest as faster parasite and vector life-cycles, contributing in particular to a rapid increase in vector population sizes [26]. In contrast, there is also the possibility of disease transmission being reduced or prevented in endemic regions due to detrimental effects on the insect vector, the parasite, and/or their interaction, and their failure to adapt rapidly enough to warmer temperatures [36–38].

In this context, understanding the thermobiology of *R*. *prolixus* and its relationship with *T*. *cruzi* infection is important, and should be extensively investigated, so that the proximate causes of the effects of temperature can be better understood. In this study, we aimed to investigate the effects that temperature has on the development and life-cycle of *R*. *prolixus* by monitoring at four different temperatures the survival, molting time, nutritional habits, and energy consumption of these insects. In addition, we followed the temporal dynamics of parasite load through the different nymphal instars and adult developmental stages of the triatomines in order to determine the impact of chronic infection by *T*. *cruzi* on vector development and resources, and how these are influenced by ambient temperature.

## Materials and methods

### 1. Ethics statement

All experiments using live animals were performed in accordance with the Fundação Oswaldo Cruz (FIOCRUZ) guidelines on animal experimentation, and were approved by the Ethics Committee in Animal Experimentation (Comissão de Ética no Uso de Animais de Laboratório; CEUA/FIOCRUZ) under the approved protocol number LW 08/20. The protocol we used is from the Conselho Nacional de Controle de Experimentação Animal of the Ministério da Ciência, Tecnologia e Inovações (CONCEA/MCTI; http://www.cobea.org.br/), which is associated with the American Association for Animal Science (AAAS), the Federation of European Laboratory Animal Science Associations (FELASA), the International Council for Animal Science (ICLAS) and the Association for Assessment and Accreditation of Laboratory Animal Care International (AAALAC).

### 2. Organisms

The *R*. *prolixus* colony used in this study originated from insects collected in Honduras in the 1990s. Insects were maintained by the Vector Behavior and Pathogen Interaction Group at the Instituto René Rachou. Experimental triatomines were fed on SWR/J mice anesthetized with intraperitoneal injections of a mixture of ketamine (150 mg/kg mg/kg; Cristália, Brazil) and xylazine (10 mg/kg; Bayer, Brazil).

*Trypanosoma* infection was performed using the *T*. *cruzi* strain Dm28c originally isolated from a naturally-infected *Didelphis marsupialis* [39]. Parasites were cultured *in vitro* by two weekly passages in liver-infusion tryptose (LIT) medium supplemented with 15% fetal bovine serum (FBS), 100 mg/ml streptomycin and 100 UI/ml penicillin [40]. In order to prevent loss of infectivity, parasites were submitted to cycles of triatomine-mice transmission every fifteen weeks.

### 3. Experimental procedures

For *T*. *cruzi* infection, a single SWR/J mouse was intraperitoneally inoculated with 200 μl of triatomine urine containing metacyclic trypomastigotes. On day 9 after inoculation, the mouse was anesthetized and exposed to 15 day-old first instar nymphs of *R*. *prolixus* until the latter were fully engorged (*n* = 104), following the protocol from [41]. The triatomines were all fed at the same time on the same mouse, whose parasitemia was assessed using Brenner’s method and found to be on average 15.48 parasites per μL of blood [42]. The first instar nymphs from the infected group ingested a mean ± SE of 3.14 ± 0.08 μL of blood, resulting in an estimated 49.6 parasites ingested per triatomine individual. Infection of the triatomines was confirmed by the consistent presence of *T*. *cruzi* in the urine/feces of the insects during subsequent blood-feeding of the following instars. The same procedures were used for the control group of triatomines blood-fed on an uninfected mouse (*n* = 104). After the first blood meal, the insects were randomly assigned to one of four thermally-isolated boxes and subsequently maintained at one of four different fixed temperatures at which they stayed until the end of the experiment: namely a mean ± SE of 24 ± 0.3°C, 26 ± 0.2°C, 28 ± 0.2°C and 30 ± 0.2°C. These temperatures were chosen based on the established preferences and tolerances of *R*. *prolixus*, which are known to develop within temperature range of 16°C to 35°C, with a preference for temperatures around 25°C to 26°C [29,43,44]. We thus selected a temperature range increasing in 2°C increments, which included the preferred range of this insect (24°C and 26°C). The upper limit of 30°C was chosen as it is the theoretical threshold after which temperature stress becomes detrimental to *R*. *prolixus* and yet still allows an assessment of realistic future environmental conditions resulting from global warming [29,44–46]. Every box had a 45 ± 5% RH and was placed in a 12:12 light-dark (LD) illumination cycle. Each box contained 52 insects, half of them infected and half of them uninfected. Each nymph was given a unique code name, and within each box they were kept individually in plastic containers (4 cm diameter x 2 cm high) covered by a piece of cloth. This ensured no contact between the individual insects and allowed us to collect data from each individual until the end of the experiment (which was 7 days after the second blood meal of adults). A piece of filter paper with feces from fifth instar nymphs of the colony was placed in each container to ensure the presence of symbionts. The triatomines were inspected daily for molting and survival. An insect was considered dead if it exhibited no response to external stimuli (e.g., tapping of the plastic container) and remained immobile while not standing on the container or filter paper surface. The insects that died during the experiment were removed from the boxes in which they had been maintained and were disposed of.

After the first nymphal instar, the insects received at each developmental stage a single blood meal on uninfected anesthetized mice 15 to 16 days after each molt. Each insect was offered such a blood meal only once per instar, and in each instance allowed to blood-feed to their full capacity. In order to ensure individual tracking, the triatomines were blood-fed in groups of six individuals, with each individual having a different one of their legs marked with a non-toxic correction fluid. The body weight of each individual triatomine was recorded both immediately before and immediately after blood-feeding, with the end of feeding determined as the moment the insects folded their proboscis and started walking. In order to account for the weight of the correction fluid used to mark the individual legs, the insects were also weighed before and after the application and drying of the correction fluid, so that the so determined weight of the correction fluid could be subtracted from the post-feeding weight measurement. The data from weighing the insects at these different time points allowed us to derive several variables: (i) the quantity of blood ingested (weight after blood-feeding–weight before blood-feeding), (ii) the total blood ingested until imaginal molting (the sum of the weights of all the individual nymphal blood meals), (iii) the blood ingestion ratio, being the number of times of their unfed weight the insects ingested in blood (blood-fed weight / unfed weight), and (iv) the retention performance, being the fraction of the blood meal weight which is retained until the subsequent developmental stage, whether it be as growth or energetic reserves. To calculate retention performance, we developed the following formula:

$$Retention\;Performance=\frac{\left(unfed\;weight(instar\;N)-unfed\;weight(instar\;N-1)\right)}{weight\;of\;blood\;ingested\;at\;instar\;N-1}$$

Where instar N– 1 is the preceding instar, at which the blood meal was consumed, and instar N is the current instar, by which the blood meal from instar N– 1 was already metabolized.

Due to limitations of the precision of the weighing scale used, accurate measurements for unfed nymphs in the first nymphal instar were not attainable. In the cases where this number was needed, we obtained an average value by weighing simultaneously all the nymphs for each treatment group used in the experiment and then dividing this combined weight by the number of nymphs weighed. All weight measurements were performed using a microanalytical balance (Shimadzu AUW220D; accuracy of ±0.1mg).

On the day after imaginal molting, adults were sexed, weighed and their length was measured using a caliper ruler. An estimation of their nutritional state was obtained by dividing weight by length, as described in [47]. These two measures of weight also allowed us to study the loss of weight that occurred between the first day after imaginal molting and the first blood-feeding of the adults 15 days later, by dividing the weight at fifteen days post-molting by the weight after imaginal molting to obtain the proportion of weight maintained. All adults were blood-fed twice with a 15-day interval between each blood meal, as described above for the nymphal instars. The experiment was ended 7 days after the last (i.e., second) blood meal of the adult triatomines. The experiment lasted 185 days from the first instar nymphs blood-feeding to 7 days after the last blood meal of the adults.

Immediately after post-blood-feeding weighing, the triatomines were placed vertically for 150 min head up in Eppendorf tubes for urine collection, for which period they stayed in a 27°C incubator. The insects were then put back in their original boxes, and returned to their appropriate experimental temperature until their next blood meal. Urine volume was measured by counting the contents of the Eppendorf with a 0.5–10 μL micropipette. Parasite concentration was determined through direct counting using a Neubauer chamber as follows: 10 μL of undiluted homogenized insect urine were loaded into the chamber, and the number of parasites within the four principal grids was counted under light microscopy. The concentration of parasites per microliter was determined by taking the average count from the four grids and multiplying it by 10, as each grid represents 0.1 μL of urine.

### 4. Statistical analysis

The effects of developmental stage, temperature, infection status (i.e., uninfected versus infected treatments), and the interaction between them on different growth- and metabolism-related variables–namely (i) unfed weight, (ii) blood ingestion ratio, (iii) molting time, and (iv) retention performance–and parasite concentration and urine production were analyzed using generalized estimation equations (GEE). GEE is a modeling approach best suited for analyzing longitudinally collected data with repeated measurements of the same samples, as is the case in our study. This method is particularly advantageous in capturing trends and variations across the entire study period, enabling the incorporation of several different possible explanatory variables and different treatments, by calculating the individual contribution to the overall variance of each of the variables/treatments studied. In the context of GEE analysis, the Wald statistic serves as a metric to assess the significance and importance of individual predictor variables in explaining the variance of the response variable. It quantifies the contribution of each variable to the overall model fit. Higher Wald statistics indicate greater importance, suggesting that the corresponding variable has a more substantial impact on the observed outcomes. Comparing Wald numbers across different variables allows for an understanding of their relative importance in influencing the dependent variable. Moreover, GEE modeling is well-suited for datasets where measurements might be missing for individual samples at certain time points, which enabled us to incorporate the previous measurements from insects that died during the experiment into our analysis.

This approach provides a comprehensive overall simultaneous understanding of how the variables interact, which is not possible through isolated and separate multifactorial comparisons for each individual time point. As a result, our graphs resulting from the GEE analysis do not incorporate symbols such as asterisks or letters to highlight statistical significance at individual time points, but instead convey the overall trends and variations present in the data, ensuring a more comprehensive and context-rich interpretation. The developmental stage of the insect was used as the mandatory time-step in the models. Additional explanatory variables were incorporated into the GEEs when their potential effect was suspected or needed to be accounted for. The sex of the triatomines was not included in these analyses as only fifth instar nymphs and adult sex was determined, which makes this information survivorship-biased. For each variable, a comprehensive range of GEE models assuming different distributions and covariance matrices were tested. The best model was selected based on the lowest quasi-likelihood information criterion (QIC). Residual normality was confirmed through QQplot examination in cases where Gaussian distribution was assumed in the model. Model fitting and QIC determination were performed using the *geepack* [48] and *gee* packages [49] in R (version 4.2.1) [50]. Effects of independent variables were extracted from the models and graphical representations were generated using the *ggeffects* [51] and *emmeans* packages [52]. The details of the structures and results of all the models generated can be found in S1 GEE Models

Effects identified in the GEE models were subsequently studied individually. This was done by creating models using only the temperature or infection status at which an interaction was observed and/or by independent statistical analysis, going more in depth on the effects at each developmental stage as this information is lacking in GEE modeling.

Differences in survival were evaluated using Cox proportional hazards regression models with temperature and infection treatments as factors. The effect of infection treatment in each individual temperature treatment was then assessed separately using the same method. The effects of temperature and infection on adult nutrition status (W/L), weight loss at the adult stage, and the total blood ingested until imaginal molting was tested using two-way ANOVA. The various correlations were tested using Type II ANOVA on linear regression models, including the effects of parasite concentration and the quantity of blood ingested in the preceding instar on retention performance, the effects of unfed body weight, preceding instar parasite concentration and quantity of blood ingested on subsequent parasite concentration, as well as the effect of infection on the quantity of blood ingested.

All numerical values presented in the text alongside confidence intervals represent means ± standard error (SE). In all statistical tests performed in this study, the level of significance was set to α ≤ 0.05.

## Results

### Growth and survival

**Fig 1:**
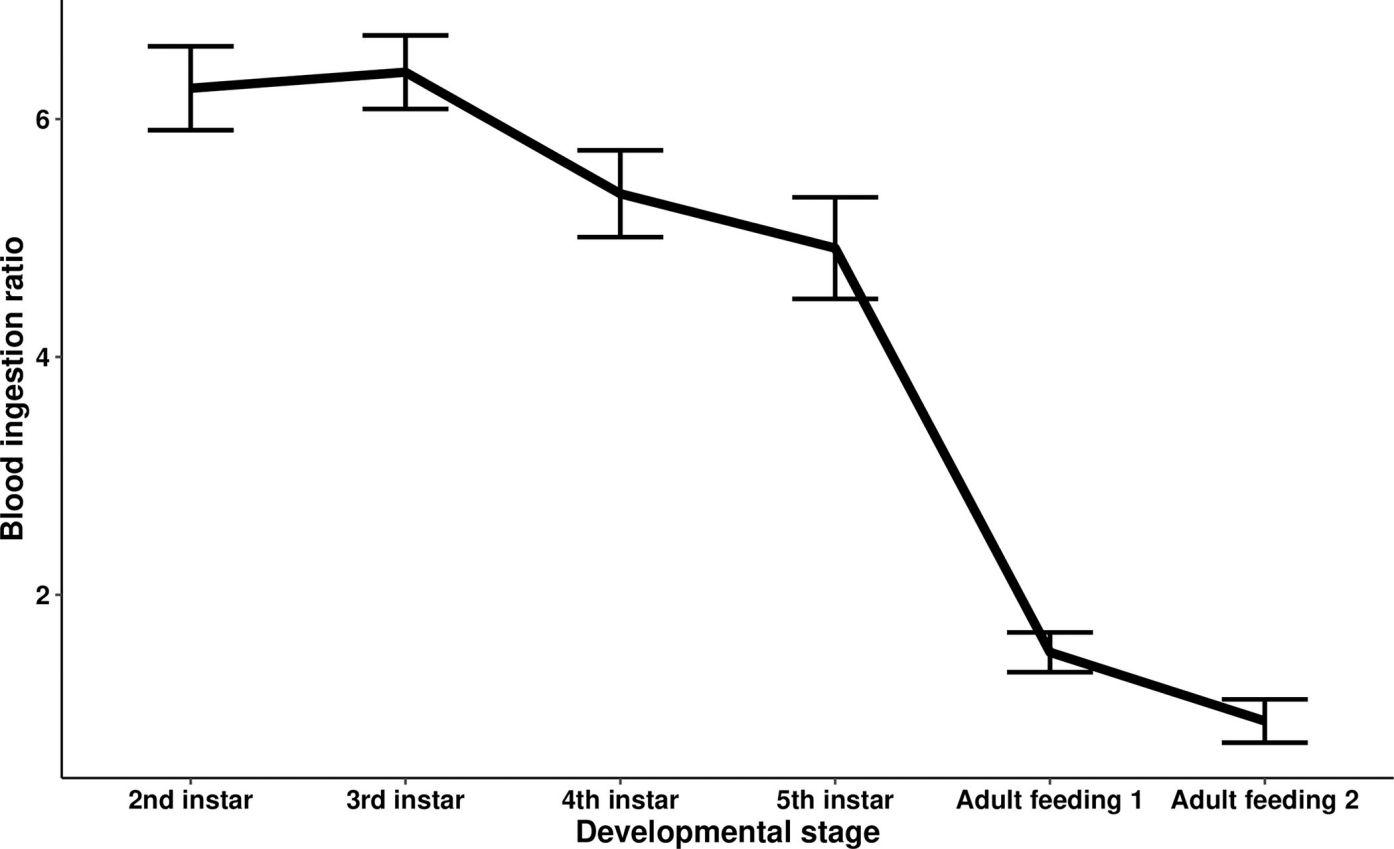
The mean blood ingestion ratio of *Rhodnius prolixus* blood-fed at different developmental stages. The blood ingestion ratio is the blood-fed body weight of each individual triatomine divided by its own unfed body weight immediately prior to blood-feeding. The entire dataset from all triatomine individuals (i.e., of every experimental group) was analyzed simultaneously controlling for developmental stage, as well as temperature and infection treatments. The blood ingestion ratio showed a significant decrease with triatomine developmental stage (Wald statistic = 24.97 *p* = 5.8e-07, *n* = 185). Error bars represent the upper and lower limit of the Gaussian confidence interval.

The GEE models showed no significant effects of temperature and infection treatment (i.e., uninfected versus infected) on the unfed body weight of the insects or on the blood ingestion ratio. Interestingly, however, developmental stage was associated with a significant reduction of the blood ingestion ratio (Wald statistic = 24.97, *p* = 5.8e-07). An appreciable decline in the blood ingestion ratio was observed between the third and fourth instars, decreasing from 6.26 ± 0.18 at the second instar to 4.91 ± 0.22 at the fifth instar. Furthermore, during the adult stage, triatomines consumed 1.52 ± 0.08 times their unfed weight during their first blood-feeding and 0.9 ± 0.09 times during their second blood-feeding. (Fig 1).

**Fig 2:**
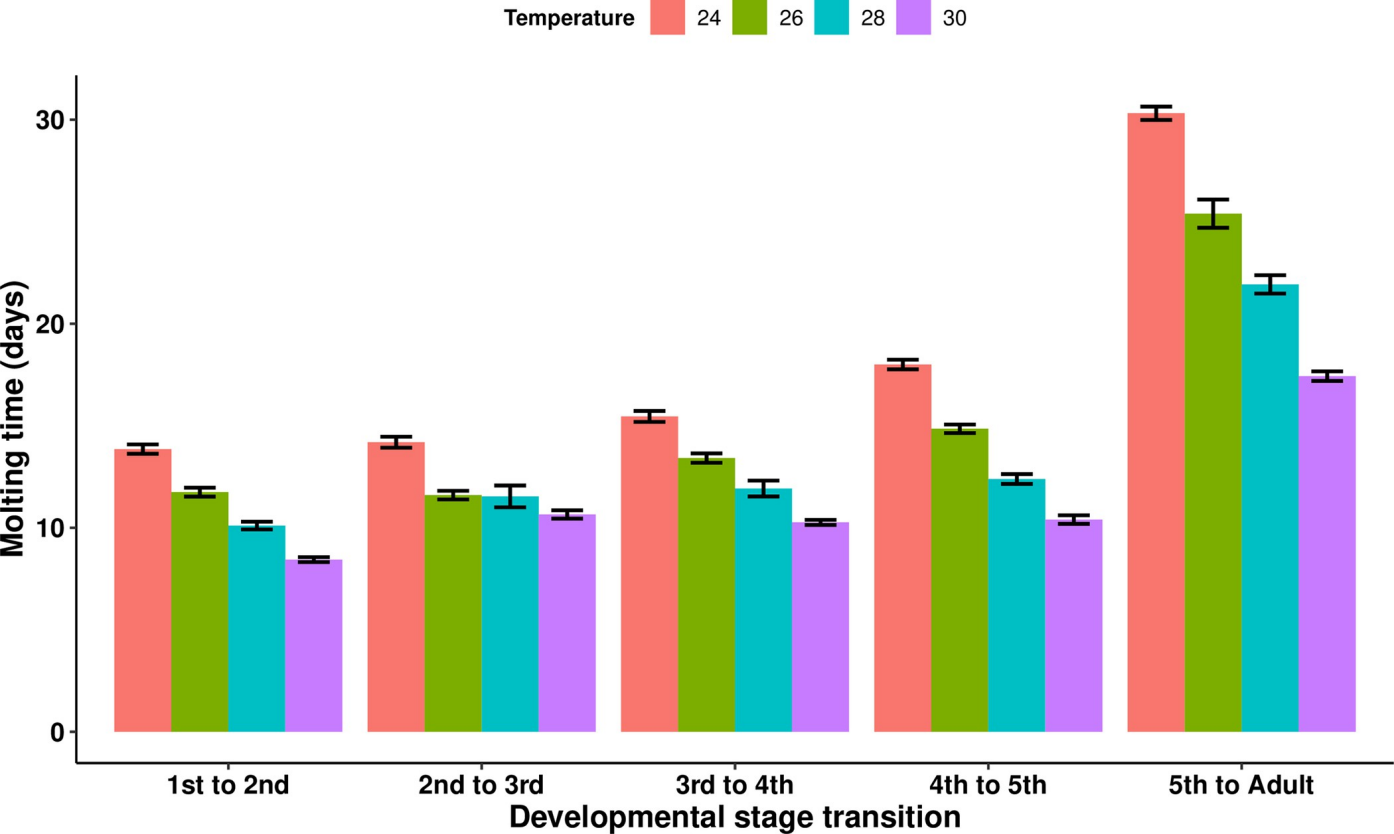
Variation in the mean molting time after blood-feeding of *Rhodnius prolixus* at different triatomine developmental stages and ambient temperatures. The entire dataset from all triatomine individuals (i.e., of every experimental group) was analyzed simultaneously controlling for developmental stage, as well as temperature and infection treatments. The duration of the inter-molt period for each developmental stage transition differed significantly between the four temperatures investigated (*p* < 2e-16 for all six comparisons between temperature treatments) and progressively increased at every developmental stage (Wald statistic = 2142.91 and *p* < 2e-16). (Overall, *n* = 197.) Error bars represent the standard error of the mean.

We analyzed the entire dataset from all triatomine individuals (i.e., of every experimental group) simultaneously for the effects of developmental stage, temperature and infection treatment on the duration of the period between molts–i.e., the “molting time”. Overall, the molting time progressively increased at every instar (Wald statistic = 2142.91 and *p* < 2e-16) and progressively decreased with higher temperatures (*p* < 2e-16 between every temperature treatment). The 30°C treatment group reached the adult stage in 116.86 ± 0.66 days, whereas the 28°C treatment group took 126.11 ± 1.11 days, the 26°C treatment group 136 ± 1.03 days, and the 24°C treatment group 151.41 ± 0.56 days (Fig 2). Infected insects maintained at 26°C took significantly longer to molt compared to the uninfected control insects maintained at the same temperature (Wald statistic = 9.3 and *p* = 0.0023), with the infected group taking 139.18 ± 1.44 days to reach the adult stage and the control group 133 ± 1.08 days. There was no significant difference in molting time between the uninfected and infected groups at the other three temperatures. These durations represent the cumulative time insects took to molt after blood-feeding, and included the experimentally-imposed 15 day periods between each nymphal molting and blood–feeding, which occurred at every instar (totaling 60 days over the lifespan of the insects), but do not include the 15 day period between egg hatching and the first nymphal blood-feeding.

Among the individuals that reached the adult stage, the different temperature treatment groups exhibited differences in their sex ratios. The two extreme temperatures had sex ratios biased towards males: at 24°C, 66% males (21 for 11 females), while the 30°C group had an even more pronounced sex ratio bias with 82% males (18 for 4 females). In contrast, the 26°C and 28°C groups had a more balanced sex ratio, with 55% (18 males and 15 females) and 48% males (13 males and 14 females), respectively. The nutritional status (W/L) was not significantly different between any of the treatment groups, whether measured one day after imaginal molt and fifteen days later, indicating comparable nutritional status upon reaching the adult stage regardless of the previous rearing temperature and infection status (i.e., uninfected versus infected) under our experimental conditions (i.e., the blood-feeding frequency used). Overall, through summing the individual volumes of each of the individual bloodmeals received up until imaginal molt, we found that the minimum quantity of blood ingested by any of the individuals that reached the adult stage with our protocol was 205.7 μL, with a mean ± SE of 398.4 ± 8.03 μL and a maximum of 614.2 μL, with no significant difference observed between any of the treatment groups.

Although the nutritional state was similar between the different treatment groups, temperature strongly affected weight loss by adults in the 15 days after the imaginal molt. The triatomines kept at 24°C had the highest weight retention of all groups with 79.35 ± 0.48%. This was significantly different from the 30°C (69.02 ± 0.62%, *p* = 8.01e-10), 28°C (75.18 ± 0.96%, *p* = 0.0113) and 26°C (77.38 ± 0,67%, *p* = 0.0107) treatment groups (*R*^2^ = 0.5322, *F*_(7,105)_ = 17.07). The adults from the 26°C and 28°C treatment groups showed similar levels of weight loss, and were both significantly different from the 30°C treatment group (*p* = 0.000154, *R*^2^ = 0.4658, *F*_(5,75)_ = 13.08 for the 26°C group; and *p* = 0.00069, *R*^2^ = 0.3971, *F*_(3,45)_ = 9.879 for the 28°C group). The 30°C treatment group had the had the highest weight loss, with an average of 69.02 ± 0.62% of weight maintained (S1 Fig). None of the temperature treatment groups showed differences in weight loss dependent on their infection status (i.e., uninfected versus infected).

**Fig 3:**
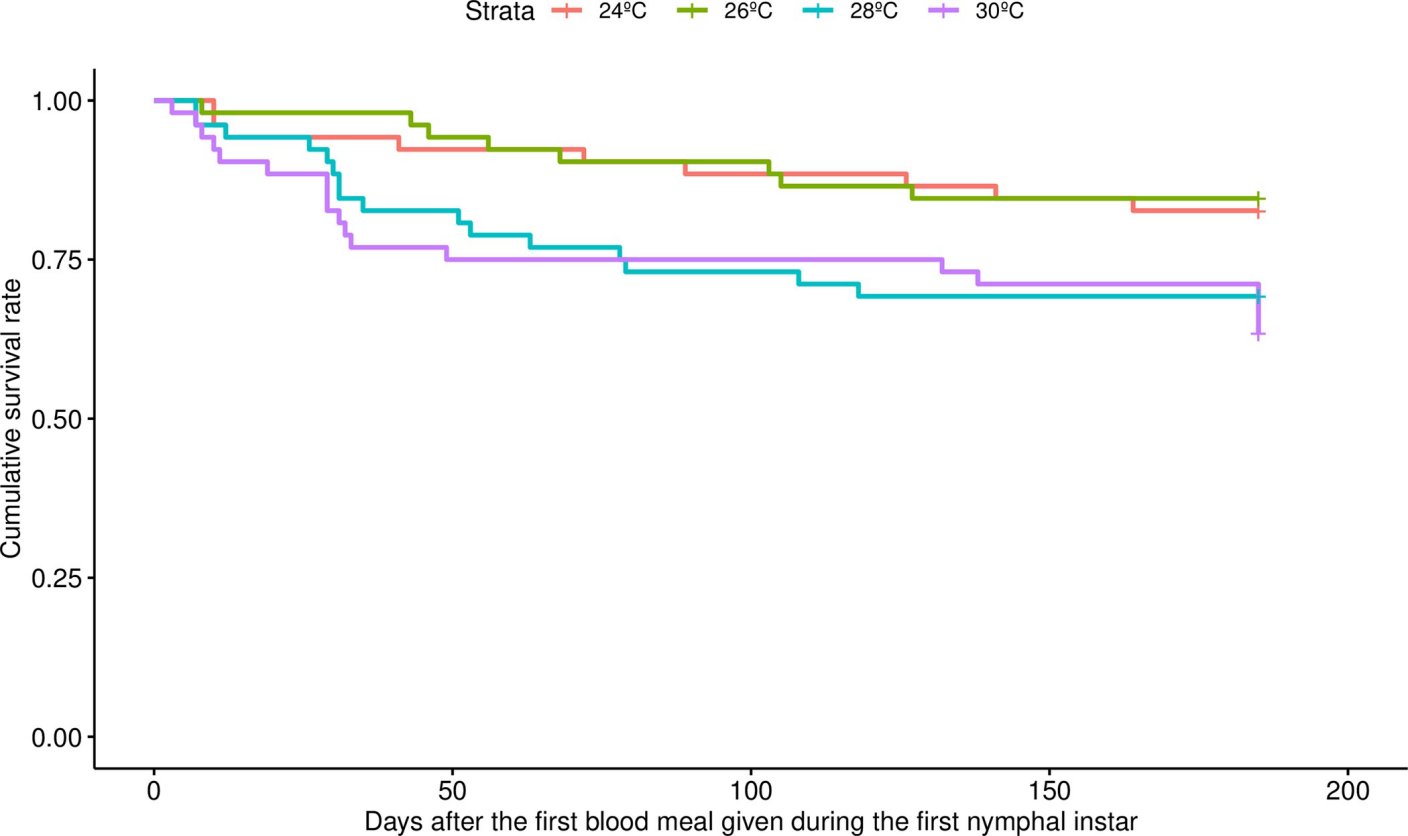
Survival rate of *Rhodnius prolixus* maintained at four different temperatures during the 185 days of the experiment. The entire dataset from all triatomine individuals (i.e., of every experimental group) was analyzed simultaneously controlling for temperature and infection treatments. The insects kept at 30°C had lower survival than those kept at 24°C and 26°C (*p* = 0.0318 an *p* = 0.016, respectively, with Cox proportional hazards regression model). Overall, *n* = 208, with *n* = 52 per temperature treatment group.

Temperature significantly affected triatomine survival, as the 30°C treatment group had significantly lower survival rates than the 24°C (*p* = 0.0318) and 26°C (*p* = 0.016) treatment groups (Fig 3). By the end of the experiment, over 30% of the insects from the 30°C and 28°C treatment groups had died (19 and 16 insects respectively, out of 52 each), while in the 26°C and 24°C treatment groups, the mortality rate was lower than 20% (respectively 8 and 9 insects, out of 52 each), without any effect on mortality of the infection treatment (i.e., uninfected versus infected) detected at any of the temperatures studied. Most of the deaths in the 30°C and 28°C treatment groups occurred within the first 50 days out of 185 of the experiment, with 12 out of 19 insects in the 30°C treatment group, and 14 out of 16 in the 28°C treatment group, dying during the first three nymphal instars, the mortality rate remaining relatively low during the later instars and adulthood.

### Retention performance

**Fig 4:**
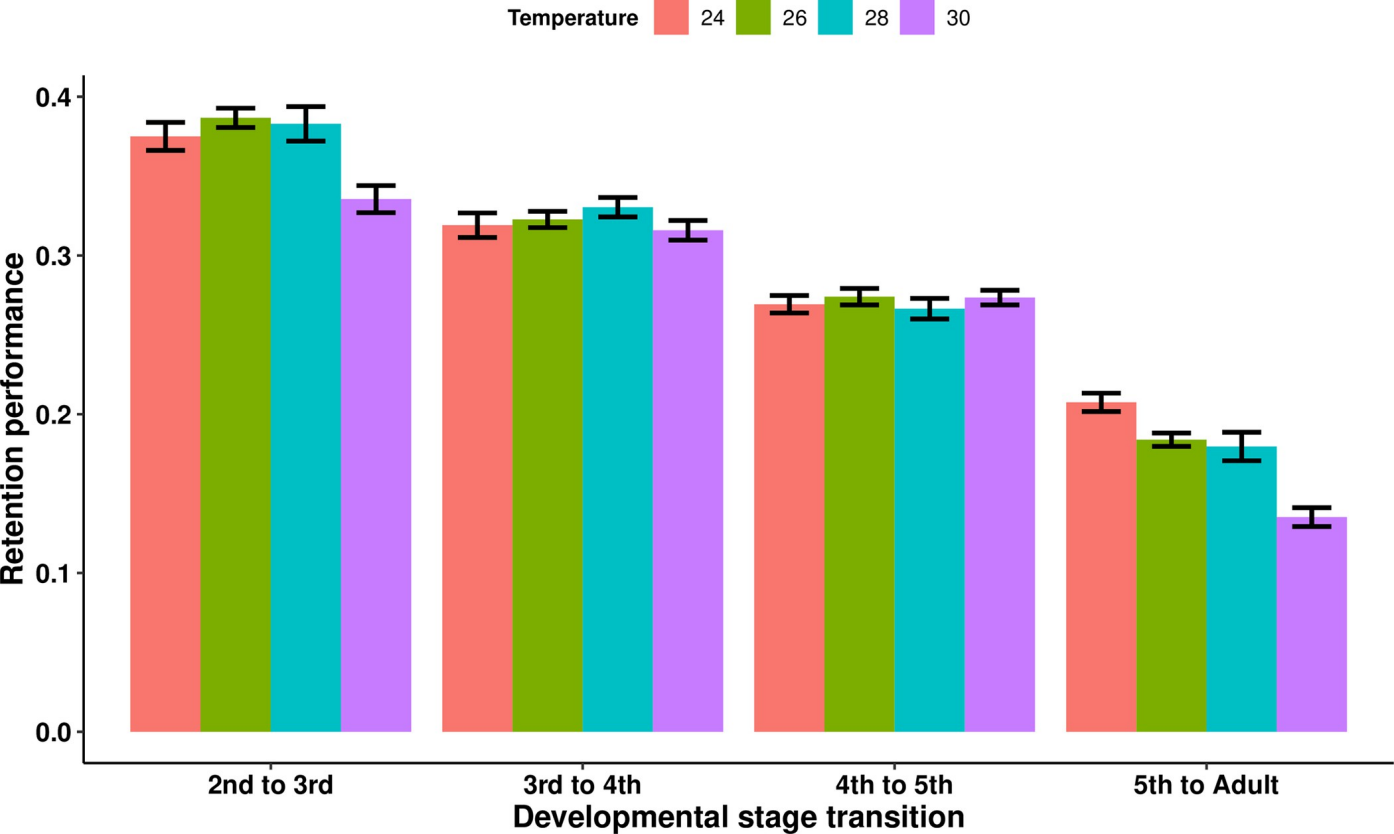
Mean retention performance of *Rhodnius prolixus* at different triatomine developmental stages and ambient temperatures. Retention performance measures the proportion of the weight of the blood meal ingested in the previous nymphal instar that is maintained as triatomine body weight in the next instar, and is calculated for each individual as the weight gained between triatomine developmental stages divided by the weight of the blood ingested by the previous nymphal instar. The entire dataset from all triatomine individuals (i.e., of every experimental group) was analyzed simultaneously controlling for developmental stage, as well as temperature and infection treatments. The triatomines kept at 30°C had a significantly lower retention performance compared to the other three temperature treatment groups (comparison with: the 24°C treatment, Wald statistic = 6.22 and *p* = 0.013; the 26°C treatment, Wald statistic = 8.72 and *p* = 0.0032; and the 28°C treatment, Wald statistic = 22.8 and *p* = 1.79e-06; *n* = 173 for all comparisons). Additionally, retention performance decreased with developmental stages (Wald statistic = 981.38 and *p* < 2e-16). Error bars represent the standard error of the mean.

**Fig 5:**
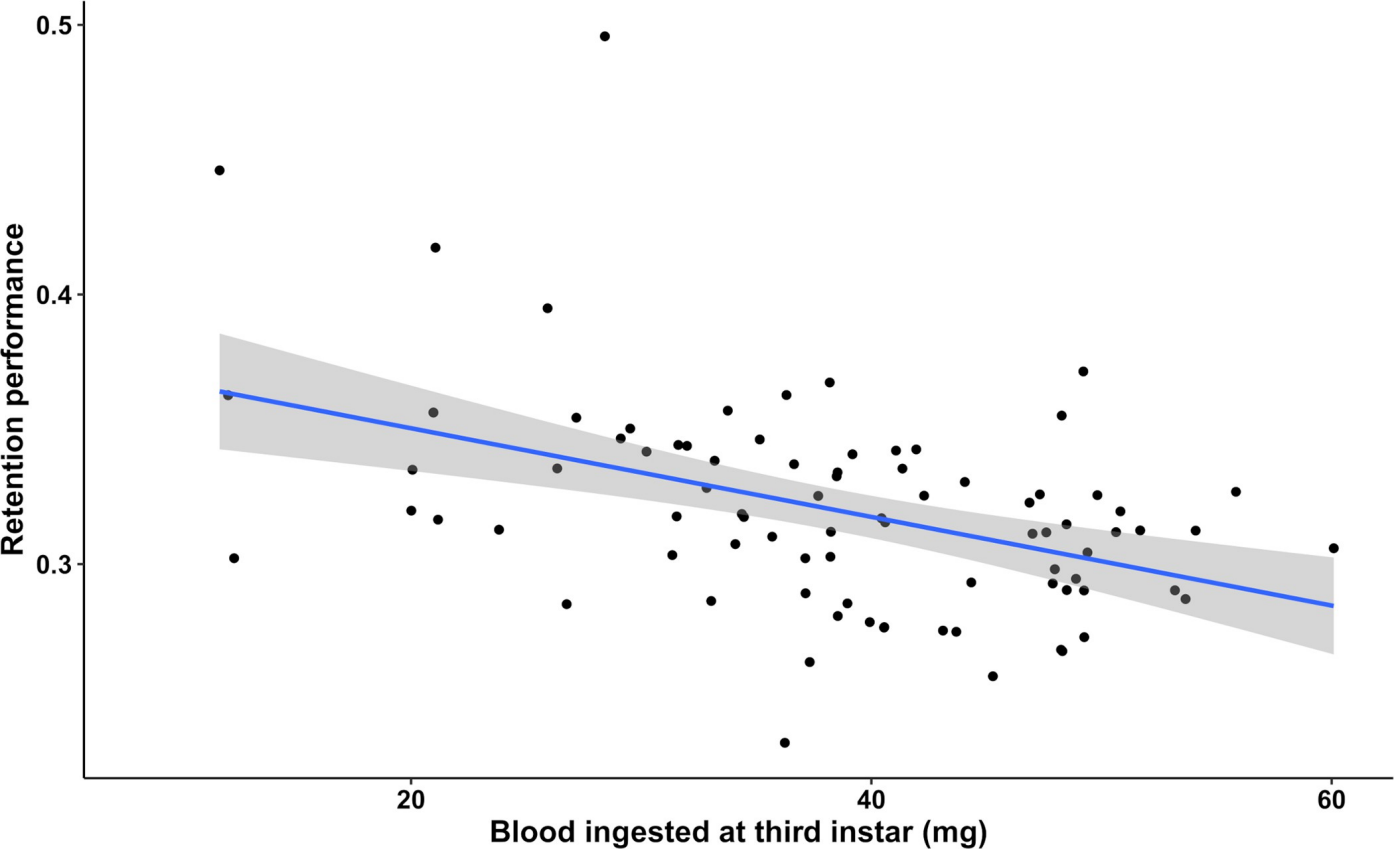
Correlation between retention performance at the fourth instar and the quantity of blood ingested in the third instar in uninfected *Rhodnius prolixus*. When the data from all four uninfected temperature treatment groups investigated were pooled and analyzed together, there was a significant linear correlation (*R*^2^ = 0.1789, *p* = 3.631e-05, *n* = 85). Grey shaded area indicates the standard error of the regression line.

We investigated the effects of developmental stage, temperature, blood meal size, and infection treatment on retention performance, i.e. the proportion of the blood meal weight from the previous nymphal instar maintained up until 15 days after molting. Overall, retention performance decreased with developmental stage (Wald statistic = 981.38 and *p* < 2e-16). Between the second and third nymphal instars, 37.08 ± 0.45% of the blood meal weight was maintained, either as body growth and/or energetic reserves, whereas between the fifth instar and the adult stage, only 18.02 ± 0.39% of the blood meal weight was retained. Temperature also affected retention performance, with insects in the 30°C treatment maintaining a significantly lower proportion of the blood meal weight than every other temperature (comparison with: the 24°C treatment, Wald statistic = 6.22 and *p* = 0.013; the 26°C treatment, Wald statistic = 8.72 and *p* = 0.0032; and the 28°C treatment, Wald statistic = 22.8 and *p* = 1.79e-06). This effect occurred mainly during the molt to the third nymphal instar and during the imaginal molt (Fig 4). Additionally, we observed, overall, a significant negative correlation between the quantity of blood ingested in the third nymphal instar and the retention performance of this blood meal at the fourth instar (*R*^2^ = 0.203, *F*_(1,164)_ = 43.1, and *p* = 6.65e-10) (Fig 5), meaning that the triatomine individuals that ingested more blood in the third instar maintained a smaller proportion of it as growth and/or energetic reserves in the fourth instar.

**Fig 6:**
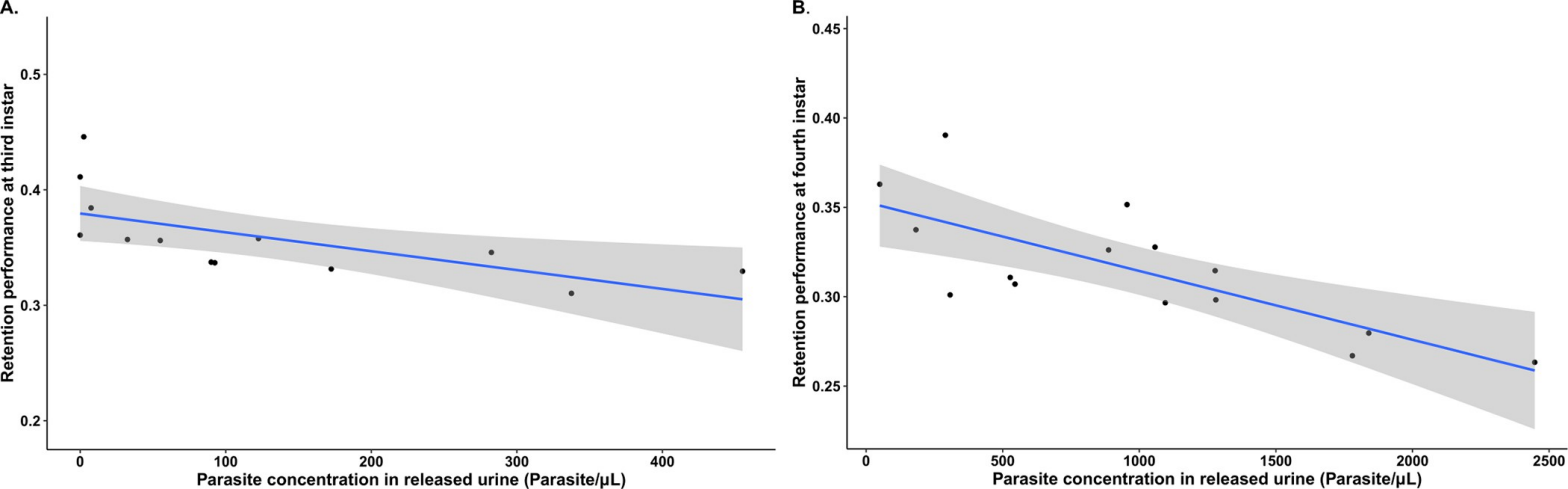
Correlation between retention performance and parasite concentration in excreted urine for *Trypanosoma cruzi*-infected *Rhodnius prolixus* kept at 28°C. A. Third instar nymphs (*R*^2^ = 0.3745, *p* = 0.0155, *n* = 13). B. Fourth instar nymphs (*R*^2^ = 0.5308, *p* = 0.001246, *n* = 15). Grey shaded area indicates the standard error of the regression line.

Although the presence or absence of *T*. *cruzi* infection did not, in general, have a significant impact on retention performance, a GEE model using all the data from all the treatment groups showed an effect on this parameter for the insects from the 28°C treatment group. Subsequent generation of another GEE model using only the data from the individual triatomines from this latter temperature treatment group, revealed that infected insects had a significantly lower retention performance compared to the uninfected control group (S2 Fig; Wald statistic = 7.78 and *p* = 0.0053). In addition, we found an association between retention performance and parasite concentration. Multiple individual simple linear regression analyses performed separately for each nymphal instar showed a significant negative correlation at the third (Fig 6A: *R*^2^ = 0.3745, *F*_(1,11)_ = 8.186, *p* = 0.0155) and the fourth (Fig 6B: *R*^2^ = 0.5308, *F*_(1,13)_ = 16.84, *p* = 0.001246) instars, indicating that for these insects those with higher parasite concentrations in their urine also tended to have a lower retention performance.

In order to confirm the effect of infection (i.e., the infected versus uninfected treatment) on retention performance, we determined the independence of infection status in the current instar from blood meal size in the previous instar, as the latter is potentially a confounding factor given our observation that the quantity of blood ingested during the blood meal of the previous instar was negatively correlated with retention performance, and positively correlated with parasite concentration, in the current instar (see the subsection “Parasite concentration in excreted urine” below for further details of these latter results). Indeed, we found that at the third nymphal instar, infected insects from the 28°C treatment group ingested more blood than those from the uninfected control group (*R*^2^ = 0.2293, *F*_(1,38)_ = 12.61, *p* = 0.00104). Consequently, in order to control for the effect of blood meal size, we created another GEE model specifically for the insects from the 28°C treatment group factoring in both the quantity of blood that these individuals ingested and their infection status (i.e., the infected versus uninfected treatment), so that we could determine the proportion of variance in retention performance that was explained by each factor. The model showed that both factors contributed to variation in retention performance, with infection status having a higher Wald number (Wald statistic = 8.18 and *p* = 0.0042) than the quantity of blood ingested in the previous instar (Wald statistic = 4.21 and *p* = 0.0401), meaning that infection status had a higher impact on retention performance than the quantity of blood ingested. Importantly, there was no significant interaction between the quantity of blood ingested and infection status in this GEE model, suggesting that these two variables are independent of one another.

### Parasite concentration in excreted urine

The experimental protocol used in this study for oral infection of the triatomines with *T*. *cruzi*–that is, a single blood meal taken from an infectious mouse during the first nymphal instar with no subsequent exposure to the parasites–was sufficient to obtain, under our experimental uninfected blood-feeding conditions and regardless of temperature, a 100.0% chronic infection success rate. In 99.0% of the individuals, this infection lasted for the remainder of either the lives of the triatomines (if the insects died before termination of the experiment) or the duration of our experiment (if the insects lived until the end of our experiment). Indeed, among the infected treatment group (*n* = 104), only one individual triatomine, from the 28°C treatment group, appeared to lose its infection some time in adulthood, after the fifth nymphal instar during which it was parasite-positive, as no parasites were detected in its urine after the two blood meals given during adulthood, nor in its intestine upon its maceration.

**Fig 7:**
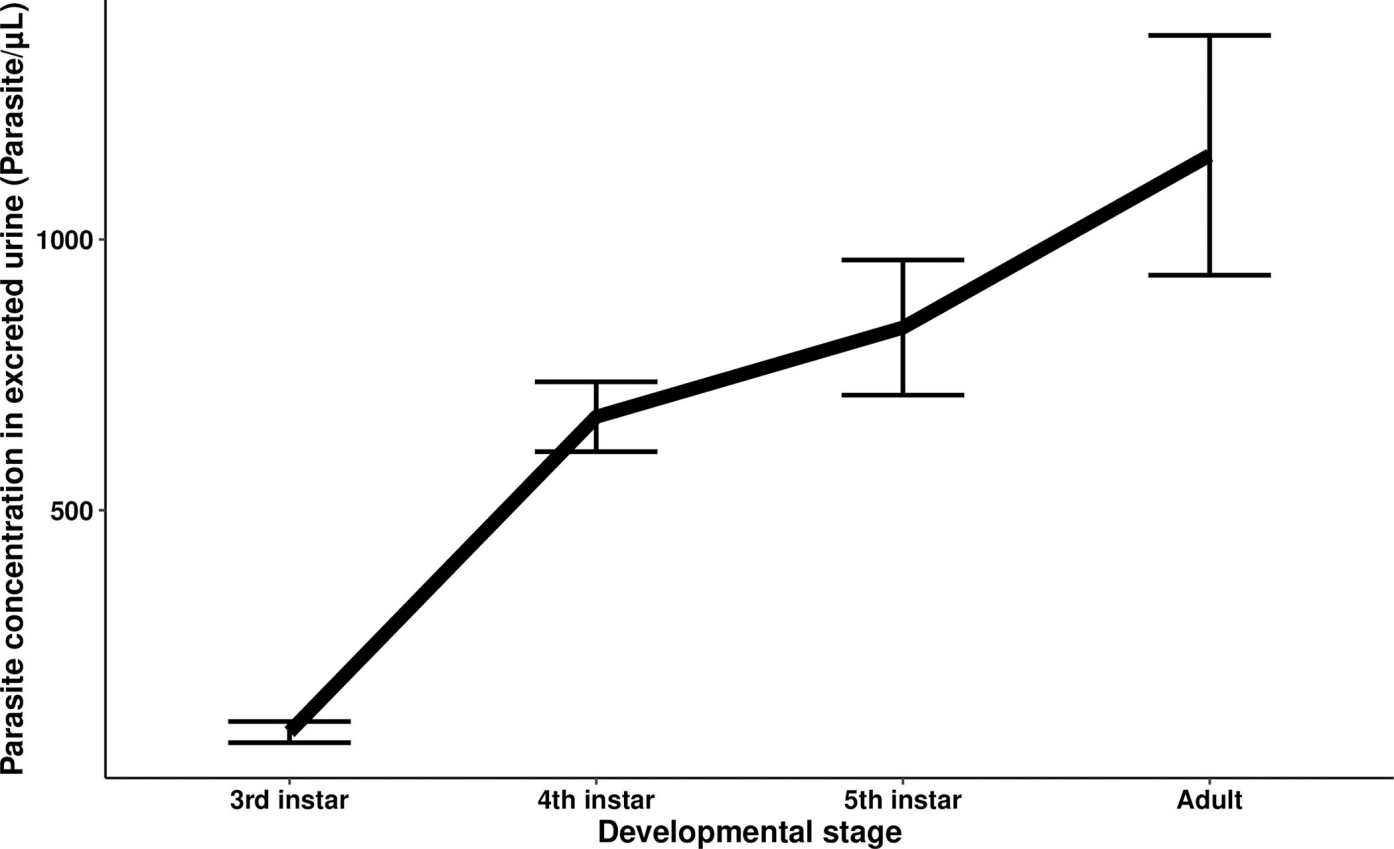
The mean parasite concentration in the excreted urine of *Trypanosoma cruzi*-infected *Rhodnius prolixus* at different triatomine developmental stages. The data from infected insects of all four temperature treatment was analyzed simultaneously controlling for developmental stage, unfed weight, and temperature treatment. The parasite concentration differed significantly between different triatomine developmental stages (Wald statistic = 64.90 and *p* = 7.8e-16, *n* = 75). Error bars represent the standard error of the mean.

**Fig 8:**
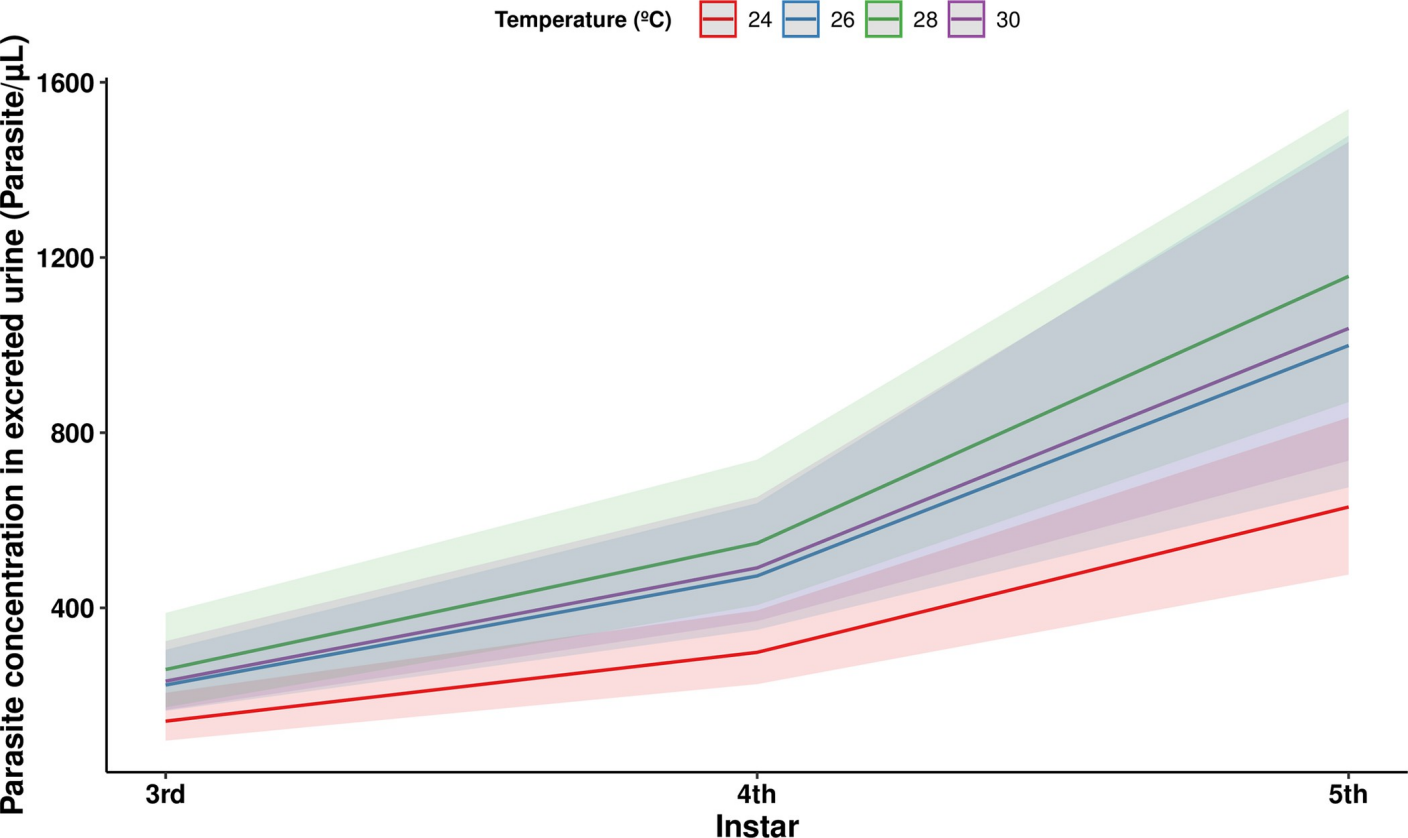
Generalized Estimating Equation (GEE) analysis showing the effect of ambient temperature on the mean parasite concentration in the excreted urine of *Trypanosoma cruzi*-infected *Rhodnius prolixus* at different nymphal instars. The parasite concentration of the 24°C treatment group was significantly lower compared to the other three temperature treatment groups (comparison with: the 30°C treatment, Wald statistic = 6.08 and *p* = 0.014; the 28°C treatment, Wald statistic = 9.53 and *p* = 0.002; and the 26°C treatment, Wald statistic = 4.64 and *p* = 0.031; *n* = 75 for all comparisons). Confidence bands represent standard error.

Using the data of the individuals from the infected groups of every temperature treatment, we analyzed simultaneously the effects of developmental stage, unfed weight, and temperature treatment on the concentration of parasites in excreted urine. The concentration of parasites in excreted urine was highly significantly associated with developmental stage (Wald statistic = 64.90 and *p* = 7.8e-16) with a rapid increase in parasite densities between the third and fourth nymphal instars followed by a slower, but continuous, increase in the subsequent fifth instar and adult stage. The mean number of parasites per μL of urine excreted increased from 90.33 ± 19.51 in the third instar to 1,155.46 ± 221.36 upon reaching the adult stage, although the inter-individual sample variation was considerable at this latter developmental stage, with some insects showing parasite concentrations exceeding 3,800 par/μL (Fig 7). Overall, when the data from all the infected experimental temperature treatment groups and all the developmental stages studied were analyzed simultaneously by GEE modelling, the insects kept at the lowest temperature, 24°C, had a lower parasite concentration compared to every other temperature treatment investigated (comparison with: the 30°C treatment, Wald statistic = 6.08 and *p* = 0.014; the 28°C treatment, Wald statistic = 9.53 and *p* = 0.002; and the 26°C treatment, Wald statistic = 4.64 and *p* = 0.031) (Fig 8). There were no other significant differences in the pairwise comparisons between the other temperature treatment groups.

The GEE model analyzing the effect of temperature treatment and developmental stage on the total number of parasites (i.e. the concentration of parasites multiplied by the quantity of urine excreted) released in the 150 min period after feeding showed that the insects from the 24°C group excreted significantly fewer parasites in total than those from the 28°C treatment (Wald statistic = 5.33 and *p* = 0.021) (S3 Fig). At the fifth nymphal instar, insects from the 28°C treatment excreted 45,568.75 ± 12,558.23 parasites, while insects from the 24°C treatment group excreted 26,606.67 ± 6,725.15 parasites. As expected from our results on parasite concentration, the GEE model showed that triatomine developmental stage also influenced the total number of parasites excreted (Wald statistic = 90.00 and *p* < 2e-16), displaying a pattern similar to the one observed on parasite concentration, with a rapid increase between the third and fourth nymphal instars, followed by a slower rate of increase between the fourth and fifth instars. Analysis of Giemsa-stained smears of the urine from randomly-selected infected individuals confirmed that the parasites excreted were predominantly of the metacyclic form, accounting for more than 90% of the observed parasites, consistent with the findings reported by [5].

**Fig 9:**
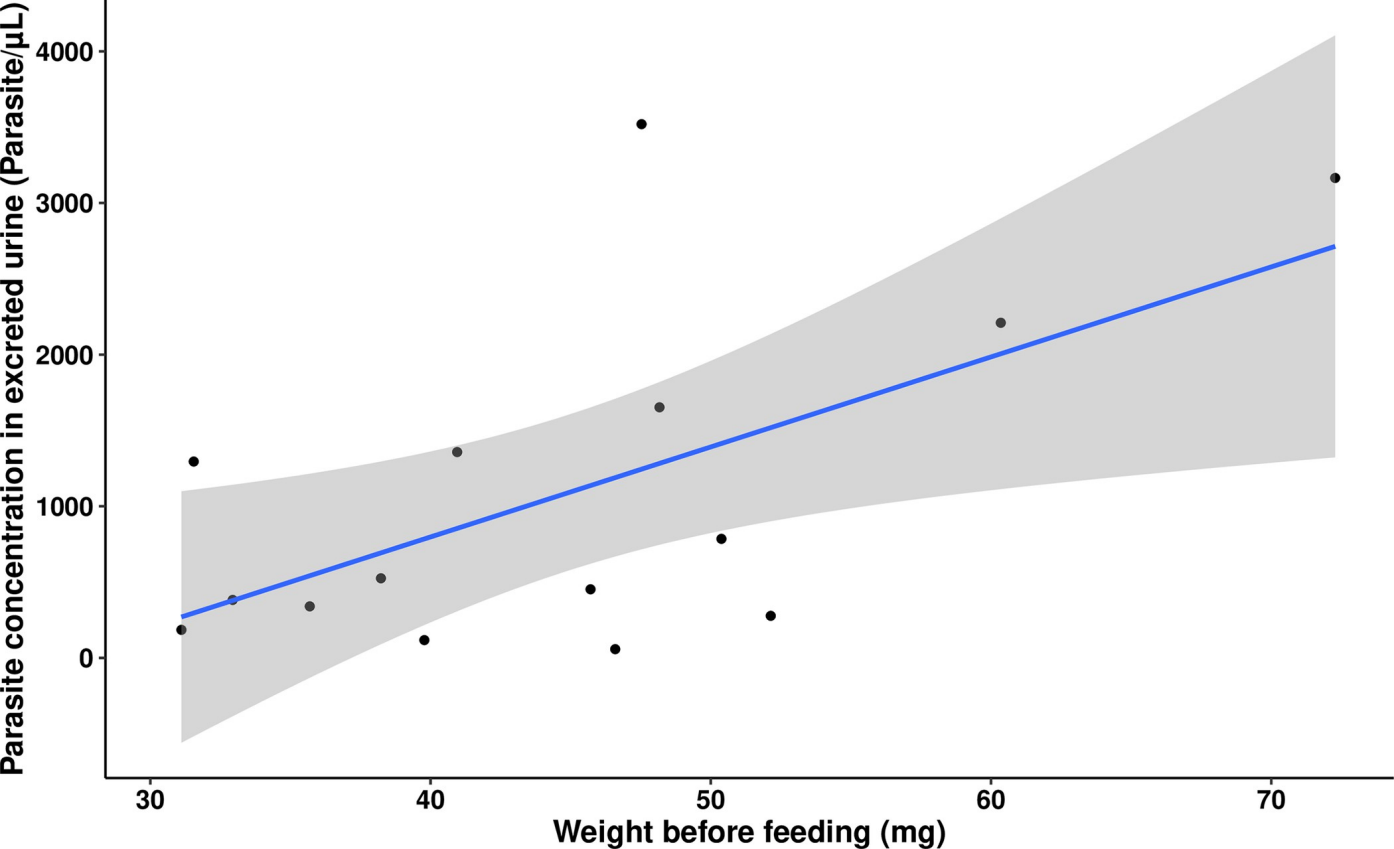
Correlation between the concentration of *Trypanosoma cruzi* parasites in the excreted urine and the unfed body weight of fifth instar nymphs of *Rhodnius prolixus* kept at 26°C (*R*^2^ = 0.313, *p* = 0.0176, *n* = 15). Grey shaded area indicates the standard error of the regression line.

**Fig 10:**
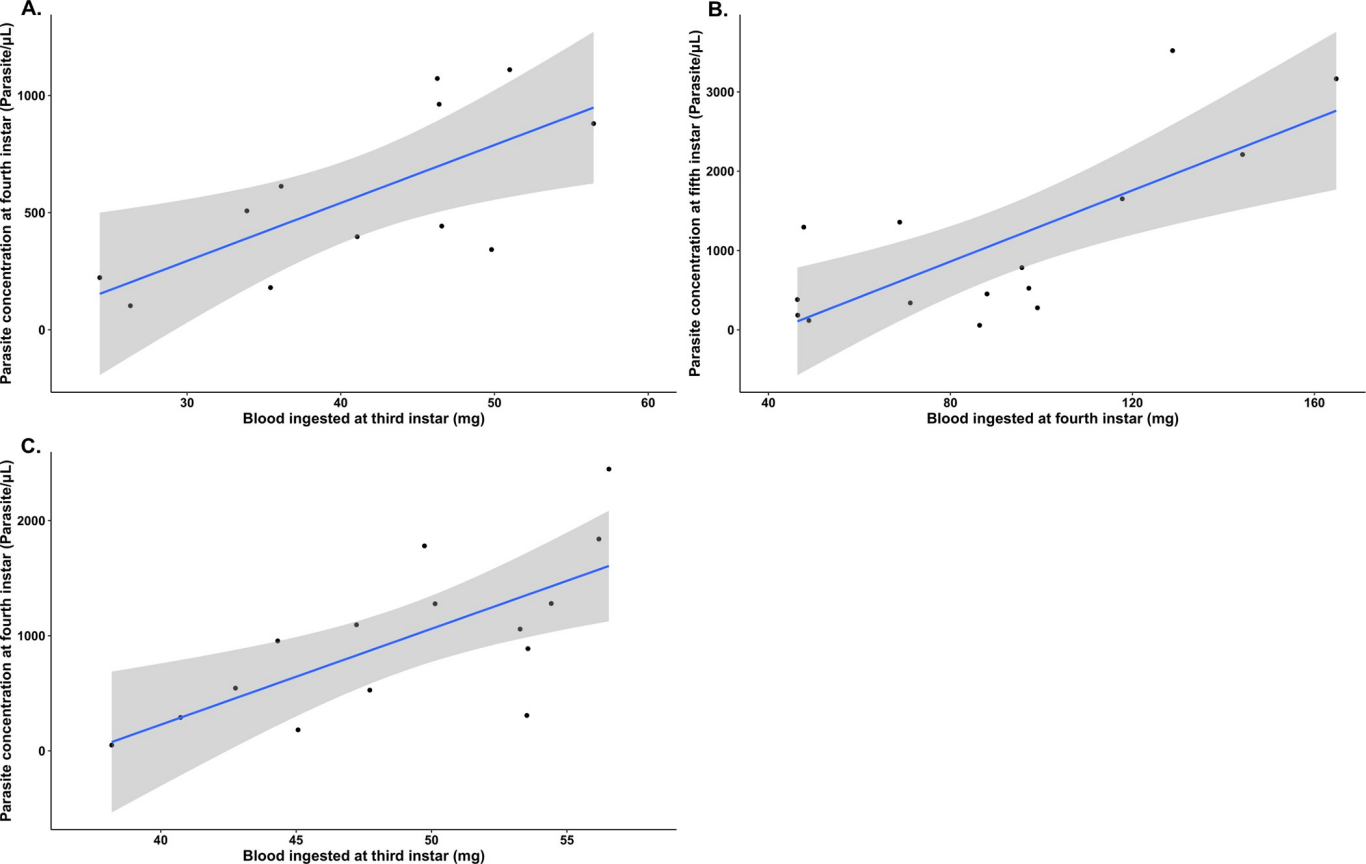
Correlation between parasite concentration in the excreted urine of *Trypanosoma cruzi*-infected *Rhodnius prolixus* and the quantity of uninfected blood ingested in the previous nymphal instar at different triatomine developmental stages and ambient temperatures. A. Fourth instar nymphs from the 26°C temperature treatment (*R*^2^ = 0.435, *p* = 0.0117, *n* = 12), B. Fifth instar nymphs from the 26°C treatment (*R*^2^ = 0.5212, *F*_(1,13)_ = 16.24, *p* = 0.00143, *n* = 15). C. Fourth instar nymphs from the 28°C treatment (*R*^2^ = 0.453, *F*_(1,13)_ = 12.59, *p* = 0.00357, *n* = 14). Shaded grey area represents the standard error of the regression line.

Our GEE model on parasite concentration also revealed an interaction between unfed body weight and the concentration of parasites excreted in the urine, but only in the 26°C temperature treatment group. A subsequent GEE model only considering the data from the insects kept under the 26°C regime showed a strong positive correlation between the weight of the insects before blood-feeding and the concentration of parasites in their urine (Wald statistic = 31.311 and *p* = 2.20e-08). Further analysis using multiple individual and separate simple linear regressions showed that although the observed values for parasite concentration excreted in the urine consistently increased with unfed weight, this effect only reached statistical significance during the fifth nymphal instar (*R*^2^ = 0.313, *F*_(1,13)_ = 7.38 and *p* = 0.0176) (Fig 9). As triatomine body weight depends on nutrition and the number and size of previous blood meals, we investigated the effect of the previous blood meal on parasite concentration. Numerous different simple linear regression models were created to determine if the quantity of blood ingested and the blood ingestion ratio of the current nymphal instar significantly predicted parasite concentration in the next instar. This analysis was conducted for every instar and temperature treatment. In every significant linear regression model, the quantity of blood ingested had the highest *R*^2^, and so was shown to be a stronger predictor of parasite concentration than the blood ingestion ratio. No significant effect was found in the insects from the 24°C and 30°C temperature treatment groups. In the 26°C treatment group, the quantity of blood ingested in the current instar positively correlated with parasite concentration at the next instar. This association was observed both for the third instar blood meal and the fourth instar parasite concentration (*R*^2^ = 0.435, *F*_(1,10)_ = 9.47, *p* = 0.0117) (Fig 10A), and for the fourth instar blood meal and the fifth instar parasite concentration (*R*^2^ = 0.5212, *F*_(1,13)_ = 16.24, *p* = 0.00143) (Fig 10B). In the 28°C treatment group, the regression models only showed a significant effect of the quantity of blood ingested at third instar on parasite concentration at fourth instar (*R*^2^ = 0.453, *F*_(1,13)_ = 12.59, *p* = 0.00357) (Fig 10C).

**Fig 11:**
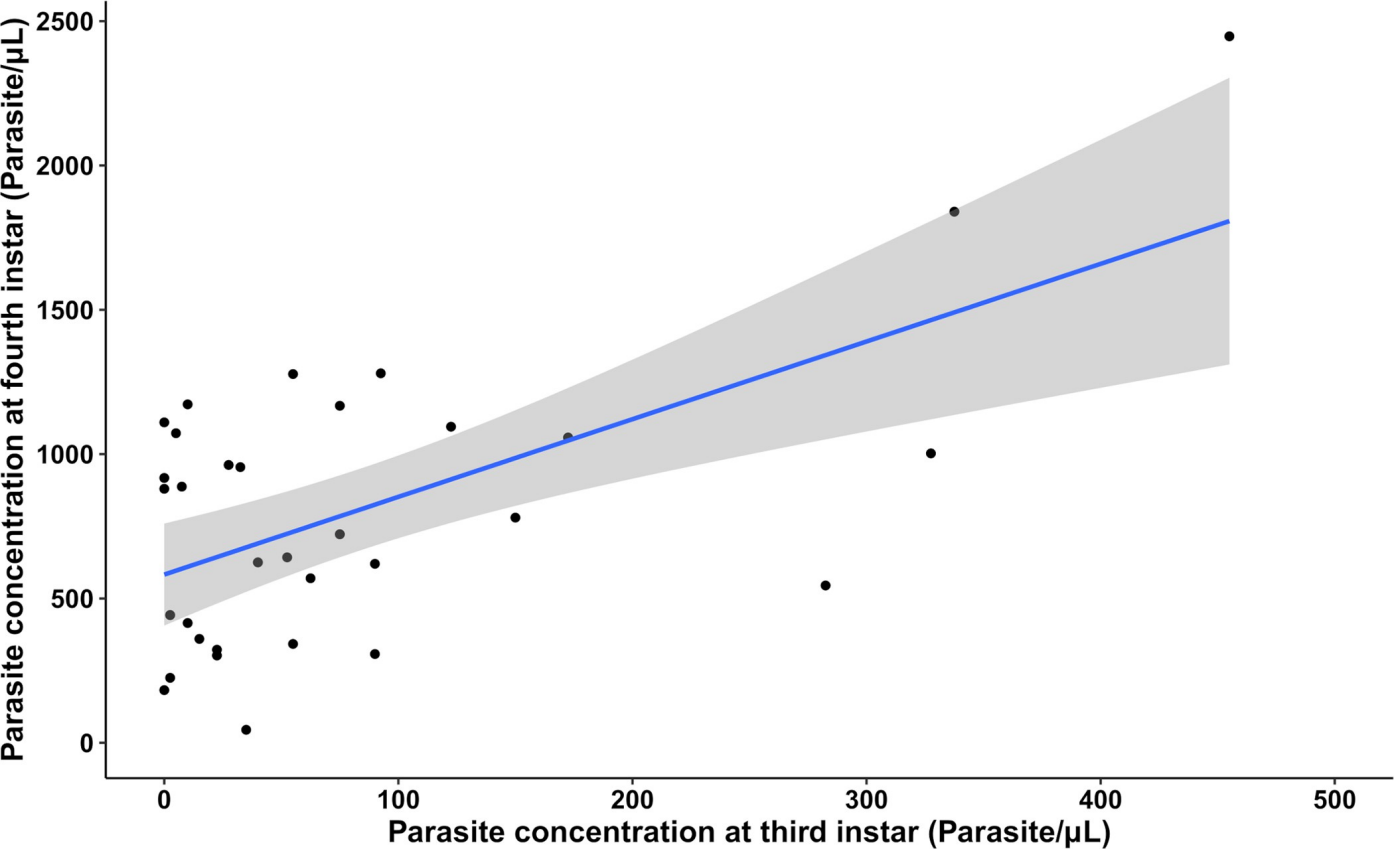
Correlation between the parasite concentration in the excreted urine of *Trypanosoma cruzi*-infected *Rhodnius prolixus* at the third and fourth nymphal instars (*R*^2^ = 0.3526, *p* = 0.000161, *n* = 33). Grey shaded area indicates the standard error of the regression line.

We also used multiple separate simple linear regression models combining the data from all infected individuals from all temperature groups to determine if the parasite concentration in the excreted urine was correlated between different triatomine developmental stages. These statistical analyses revealed that there was a significant positive linear correlation between parasite concentration at the third nymphal instar and the parasite concentration at the fourth instar (Fig 11; *R*^2^ = 0.3526, *F*_(1,13)_ = 18.43, *p* = 0.000161). However, there was no significant correlation between the parasite concentrations observed at the fourth and fifth nymphal instars suggesting that the parasite load does not always carry over between different triatomine developmental stages.

### Urine excretion and diuresis

The quantity of urine excreted in the first 150 min of diuresis by the insects following each blood meal did not show any significant effect of temperature and infection status (i.e., uninfected versus infected treatment) in the GEE models. The two variables explaining most of the variance in the quantity of urine excreted were developmental stage (Wald statistic = 1,343.851 and *p* < 2e-16) and the blood ingestion ratio (Wald statistic = 17.962 and p = 2.25e-05), which also did not themselves differ significantly between treatments (i.e., infection status and temperature). The quantity of urine excreted was highly variable, and a certain threshold quantity of blood apparently needs to be ingested in order to initiate the process of diuresis, as is clear from graphical representation (S4 Fig). Up until the fourth instar, insects required approximately twice their unfed body weight in ingested blood in order to initiate urine excretion, while fifth instar nymphs and adults only needed the equivalent of their unfed weight in ingested blood in order to trigger diuresis.

## Discussion

In this study we aimed to investigate how temperature and infection affect the *R*. *prolixus* life cycle, as well as to obtain a better understanding of the effects of ambient temperature on the multiplication of *T*. *cruzi* within its triatomine vector. Through gathering our data for each insect individually, we were able to analyze novel variables, such as retention performance, which quantifies the proportion of the weight of the blood meal which is maintained until the next nymphal stage, as either body growth and/or energetic reserves. This allowed us to gain new insights into the *R*. *prolixus* life cycle and its energy consumption, and to determine the effects–if any–of ambient temperature and *T*. *cruzi* infection upon them.

Consistent with previous findings [53,54], we found that an increase in temperature decreased molting time, thereby attaining sooner the adult–and so reproductive–stage. However, the same increase in temperature also had an impact on insect survival, in that the groups maintained at the two highest temperatures showed increased mortality when compared to the groups maintained at the two lowest temperatures. In the former two groups, the majority of deaths occurred in the first three nymphal instars, suggesting that during the earliest instars, insects may be more vulnerable to temperature and exhibit higher mortality rates as a result. This vulnerability to higher temperatures of earlier nymphal instars could stem from a lower resistance to water loss and nutrient depletion due to their smaller body size, and so a higher area to volume ratio, especially of the first instars [45]. It is also possible that the weakest individuals, which would have died at any instar, experienced rapid mortality during the initial phases of the experiment. There probably exists a threshold temperature above which the adverse effects of temperature on individuals outweigh any benefits of accelerated metabolism, and, after which, the duration of the inter-molt time will not get any shorter. Our results indicate that this hypothetical threshold is higher than a constant temperature of 30°C, as previously observed in other triatomine species [24,46]. Overall, these results indicate that higher temperatures should, in general, have two opposing effects on triatomine population growth: (i) reducing the inter-generational time, through decreasing the inter-molt period, and thus decreasing the time required to reproduce, while also (ii) increasing mortality rates. The elevated mortality rate under high-temperature conditions could potentially exert an evolutionary selection pressure favoring the temperature-resistant individuals. The implications of these effects on the population dynamics of *R*. *prolixus* in response to global warming remain uncertain without the application of quantitative ecological modeling. Such analyses are crucial in understanding the future evolution of populations of *R*. *prolixus* in the niches that this triatomine species currently occupies, but also on potential future range shifts–both geographical and ecological in environmental niche space–in the *R*. *prolixus* distribution, which would directly affect the transmission risk of *T*. *cruzi* as well.

In addition to temperature, molting time was also affected by *T*. *cruzi* infection, as infected insects took significantly more time to molt than the uninfected control group at 26°C. This effect has already been described [13,14] across temperatures ranging from 21°C and 30°C, and using different strains of *T*. *cruzi*, suggesting a possible difference in temperature sensitivity among different parasite strains with regard to triatomine host molting time. Delayed molting increases the time before the next blood meal, which has been shown to be detrimental to both parasite development and maintenance of the infection within triatomines [3]. Therefore, a prolonged inter-molt period could result in decreased parasite population sizes within triatomine hosts, although in our laboratory setting parasite infection rates were not affected at 26°C, the temperature at which, in our study, the inter-molt period was significantly affected. In order to further elucidate the causes of, and relationships between, blood-feeding, temperature, the inter-molt duration and parasite infection, further investigation is needed on how longer fasting periods affect parasite development and infection loss within triatomines at different temperatures. This would provide valuable insights, as we did not find any effect of parasite infection on triatomine survival, although it has previously been described [14,17,22,55], but infected triatomine mortality rates increased when compared to their uninfected counterparts only after a longer fasting period than in our protocol.

Interestingly, the treatment groups exposed to either 30°C or 24°C temperature regimes showed sex ratios skewed towards males, while no sex ratio bias was observed at the other temperatures studied. Although an intriguing result, because our experimental protocol did not allow us to know the sex ratio after allocation of the first instar nymphs into their different temperature groups at the start of the experiment, we cannot affirm that the observed sex ratio biases were due to higher mortality in females held at more extreme temperatures. As only the sex of fifth instar nymphs and adults was determined, this would create a survivorship bias, since only the insects that survived until these developmental stages would be considered in the statistical analyses. Therefore, any data in which the sex of individuals was thought to have a non-negligible effect was excluded altogether from our analyses. For example, adult blood-feeding data is absent from most of the statistical tests, as females ingest more blood than males at this developmental stage in order to support egg production [56].

In our experiment, a single infective blood meal during the first instar produced a 100% infection success with 99% of infections persisting either throughout the remaining lives of the triatomines or until the end of the experiment. This means that under our experimental protocol, in which the triatomines received sufficient uninfected blood meals throughout their development and adulthood to be free of nutritional stress, we were able to obtain a chronic infection of *R*. *prolixus* by *T*. *cruzi* using only a single oral infection event. This could be why we did not observe an effect of parasite infection on blood ingestion ratio, which has been shown in previous studies, where triatomines from the infected treatment group were exclusively fed on infected murine hosts [13]. These contrasting observations from different studies could be because of the lower quality of infected blood, or some additional stress due to multiple reinfections. In fact, it has been shown that chronic infection does not increase the quantity of blood ingested by *Triatoma infestans* [57], which is consistent with our results using *R*. *prolixus*. Regardless, we have been able to report the first description of the temporal dynamics of parasite concentration in excreted urine across the different developmental stages of individually followed insects, from the moment when chronic infection was first initiated by a single oral exposure with the parasite during the first nymphal instar through the subsequent four nymphal instars and up until adulthood.

Parasite concentration in the excreted urine increased at every studied stage of triatomine development, although the rate of increase appears to be higher between the third and fourth instars than the later developmental stages. The apparent slower rate of increase in parasite concentration in the later developmental stages suggests the existence of a limiting factor governing parasite population growth within individual triatomines. In [3], the authors showed that most of the parasites reside attached to the rectal pads of the rectal ampulla of the triatomines, and proposed that the available space on the luminal surface of this part of the intestine could be such a limiting factor. However, our findings challenge this idea, as the shape of the curve for parasite concentration for the insects kept at 24°C, which had the lowest concentration of parasites, was similar in shape, although lower in absolute magnitude, to that of the insects kept at the other temperatures, implying that spatial constraints on the carrying capacity of the surface of the rectal ampulla do not explain the lower densities of parasites observed at 24°C (assuming that intestinal size does not vary with temperature). Our results show that temperature is an important limiting factor for the rate of parasite multiplication up until approximately 24°C, indicating that the rate of parasite multiplication will be at its highest level throughout the range of elevated environmental temperatures expected to be induced by climate change [27], while other factors, such as nutritional resources quantity and/or availability, may restrain parasite population growth.

Indeed, the fact that the rate of parasite growth was only affected by temperature up to 24°C is very different to observations found *in vitro* using culture media, where increases in temperature above 24°C result in increased multiplication rate [14]. Importantly, blood meal size positively correlated with parasite concentration in the excreted urine of subsequent nymphal instars in the insects from the 26°C and 28°C treatment groups. This indicates that a higher quantity of resources positively impacts parasite development, meaning that nutrient availability would be an important limiting factor of parasite multiplication at these temperatures, even at the high frequency of blood meals used in our experimental protocol. At 26°C and 28°C, we also showed a positive correlation between the parasite concentrations of consecutive nymphal instars, indicating that parasite loads are proportionately maintained between different successive triatomine developmental stages, at least at these temperatures under our experimental conditions. A similar correlation was found between the third and fourth nymphal instars, when combining every temperature treatment, but not between the other instars, possibly because the highest increase in parasite concentration appears to have occurred between these two instars. While it is logical to expect in chronic infections a correlation between the levels of parasite concentration in successive triatomine developmental stages, it is intriguing that in the 30°C treatment group this correlation was not observed, nor was there any correlation between parasite concentration and blood meal size at this temperature. In the insects maintained at 30°C, other factors, likely related to higher metabolic rates of either the parasite and/or the triatomine host, may simultaneously limit parasite multiplication, and cause it to remain at levels comparable to those observed in the 26°C and 28°C groups.

Furthermore, *T*. *cruzi* infection negatively affected the capacity of the insect to convert blood into growth and energetic reserves, as inferred through the index of retention performance, and this effect was proportional to the parasite load. We consistently observed in our data lower retention performance when parasite load was higher, although this only reached statistical significance at 28°C, one of the treatment groups in which the insects were shown to be most infected. This supports the hypothesis of the existence of an impact of parasite infection on the energetic resources of the insect, which has been previously suspected, but never formally demonstrated [16,19,58], possibly because this effect is not of sufficient magnitude to induce significantly detectable weight differences between uninfected control and infected insects. A reason for not detecting an impact of parasite infection on triatomine weight appears clearly in our data, as the insects with the highest infection levels, and thus presumably suffering the greatest cost of the presence of *T*. *cruzi*, also ingested the greatest amount of blood during the preceding instar, effectively enabling them to compensate and buffer through their diet any effect of the infection on weight. As already mentioned, most of the parasite population is located in the rectal ampulla [3,5,59]. This makes it unlikely that the effect of infection on retention performance is the result of competition between the parasite and the host triatomines, as the content of the intestines at this point is destined to be excreted by the insect [60], although parasite-host competition in those insects that also maintain parasite populations within their posterior midguts, where parasite interference with host absorption and/or utilization of the intestinal contents may occur, cannot be ruled out. The decrease in retention performance observed in infected insects might represent the cost of immune defense of the insect, although the extent of this reaction in the case of *T*. *cruzi* infection is still debated [61–64]. It could also be attributed to the perturbation of the other microbiota of the insect induced by the presence of the parasite, as such perturbation during the early weeks of triatomine infection has been described in previous work [10–12]. However, the long-term effects of this initial perturbation of the microbiota within the context of a chronic *T*. *cruzi* infection has apparently not previously been studied. The loss of retention performance also likely includes the metabolic cost of parasite multiplication and differentiation. Regardless of the specific source of this effect, our results clearly indicate that the presence of the parasite imposes a metabolic cost on the insect, and the extent of this cost increases proportionally with parasite load. However, this metabolic cost did not cause higher mortality of triatomines under our experimental conditions. Consequently, its importance for *T*. *cruzi* transmission might lie in factors not examined in this study, such as the diminished fertility of infected triatomines and their increased susceptibility to starvation induced by this stress [14,16,17,19,22]. Considering our analysis of blood meal size reported here, our results indicate that the larger the size of the uninfected blood meals given for triatomine nutrition, and the higher the parasite load of the individual triatomines, the lower the proportion of this nutritional blood meal that contributes to growth and energetic reserves, with the potentiating effect that larger blood meals result in a higher parasite intensities in the subsequent developmental life cycle stage of the triatomines.

Overall, retention performance showed a consistent and gradual decrease with developmental stage. In order to understand this phenomenon, it is necessary to question what comprises the body weight loss that occurs between the different developmental stages of triatomines which is represented by retention performance. As described in [65], a portion of weight loss occurs during diuresis immediately after blood-feeding, which partially explains why individual triatomines that ingested more blood had a lower retention performance in our models, as they produced higher volumes of urine. Another source of loss would be evaporative water loss through the cuticle, however it is known that insects tend to get more resistant to dehydration with progressing developmental stages due to their increase in body size [65]. In addition, the ability of *R*. *prolixus* to retain water is higher than that of other species of blood-feeding insects [65], suggesting that water loss is unlikely to be the cause of the stage-dependent decrease in retention performance we observed. As we have shown here, the blood ingestion ratio also decreases with developmental stages, which could again be an important factor to consider, as it means that, in comparison to earlier developmental stages, the insect would have proportionally less resources, and therefore a reduced capacity to store energetic reserves. Finally, molting is known to be an important weight loss event during the earlier instars of triatomines, and may become more costly with progressing developmental stages [66].

Another interesting effect that we observed here is that temperature also affected retention performance, with the individual triatomines from the 30°C group having a lower capacity to maintain weight until their next developmental stage. This impact of temperature was further confirmed by the comparison of weight loss during the 15 day period following the imaginal molt. It is expected that higher temperatures should induce faster metabolism [67], increased locomotor activity [68,69], and, notably, a higher rate of water loss due to the increased permeability to water of the *R*. *prolixus* cuticle at temperatures above 30°C [70,71]. These factors collectively will lead to accelerated weight loss and diminished retention performance in *R*. *prolixus*. Nevertheless, the diminished retention performance observed at 30°C did not result in a lower nutritional state (W/L) at the adult stage, most likely because the frequency of blood-feeding in our experimental protocol was sufficient for the maintenance and development of the triatomines under our experimental conditions. Future investigation of the impact of fasting time on retention performance in insects maintained under the same temperatures will be essential for determining if, and how, the index of retention performance can serve as an informative life-history trait in future studies of triatomines.

Most domestic human *T*. *cruzi* infections happen *via* contact with infected triatomine feces excreted during their blood–feeding [72], which contain the infective metacyclic trypomastigote forms of the parasite [3]. Given this unique mode of infection, investigating the diuresis of triatomines also provides important information about its vectorial capacity. Previous studies have shown that *T*. *cruzi*-infected triatomines start excreting urine sooner, and at a faster rate, than uninfected control triatomines [57,73]. However, in our study, we did not observe any effect of infection status (i.e., uninfected versus infected treatment groups) on urine excretion, possibly because urine was collected for a relatively long period of time (such that any differences in the speed of onset and/or rate of urinary excretion would have been masked). This suggests that the faster onset of urine excretion in infected insects does not imply that more urine will be excreted during the totality of the period of diuresis which follows blood-feeding. It is worth mentioning that while these findings are relevant for vectorial capacity, they only take into consideration the route of *T*. *cruzi* transmission to humans associated with diuresis. However, this is only one of the possible modes of *T*. *cruzi* transmission, as, in the field, and in most recent epidemiological outbreaks, *T*. *cruzi* infection of humans has occurred through oral ingestion by humans of plant foods contaminated with *T*. *cruzi*-infected triatomines [74–77].

As our experimental protocol focused on insects subjected to a forced fixed temperature regime, it does not account for the other factors in the field known to affect the way in which insects relate to ambient temperature and how they this may be altered by climate change. Such factors include temperature preference [24,29], microclimates [69,78] and seasonal variation [79]. Indeed, another characteristic of climate change is a greater range of variation of in temperatures between seasons, although temperatures increase overall. While our study does not account for such environmental variation, the findings we present can establish a theoretical framework for hypothesis development. Based on our results, we expect that during warmer periods a broader range of seasonal temperature variation could induce accelerated triatomine development, and faster egg production, coupled with increased insect mortality rate. In colder periods, a deceleration in the development of the insect may be observed, potentially mitigating the effect of the acceleration during hotter periods. Consequently, we suggest that the overall life cycle of the insect may remain relatively unaffected by a broader seasonal temperature range, but could be accelerated by the general increase in temperatures. However, the potentially higher mortality rate of triatomines in the hottest seasons, and the loss of synchrony between seasons and insect generations, could pose challenges to the overall stability of triatomine populations. It is equally important to emphasize that climate change extends beyond global warming. Factors such as the elevation of CO₂ levels and alterations in relative humidity are also crucial abiotic components inducing physiological and behavioral changes, alongside temperature variations [26–28].

In summary, in this study we provide comprehensive insights on the intricate effects of both temperature and *T*. *cruzi* infection on the developmental life cycle of *R*. *prolixus*. We acknowledge the inherent limitations of laboratory colonies in terms of genetic variability, particularly with regard to variation in the life cycle of these insects. Therefore, the applicability of the effects reported in this study necessitates validation through further investigation involving sylvatic *R*. *prolixus*. Our results highlight the complex interaction between triatomine nutrition and the development of *T*. *cruzi* in the intestine of these insects, as both factors affect each other and are interrelated. We have also demonstrated that even relatively minor temperature differences were enough to significantly impact the temporal dynamics of *T*. *cruzi* infection, and we have proposed several hypotheses based on our results of the possible underlying explanations for these effects. Furthermore, our findings demonstrate that although detrimental, higher temperatures do not impede the development of either the insect or the parasite. This suggests that the anticipated effects of global warming are unlikely to impede the transmission of *T*. *cruzi*.

## Supporting information

S1 GEE Models The structures and results of the GEE models created and discussed in this study. (DOCX)

S1 Dataset The complete dataset produced and analyzed in this study. (XLSX)

S1 Fig Graphical representation of the proportion of weight maintained 15 days after imaginal molt, at 4 temperatures. (TIF)

S2 Fig Graphical representation of the GEE extracted effect of infection on retention performance in insects from the 28°C. (TIF)

S3 Fig Graphical representation of the total number of parasites excreted at several life stages at 24°C and 28°C. (TIF)

S4 Fig Graphical representation of the quantity of urine excreted depending on blood ingestion ratio at five different life stages. (TIF)
