# Supplementary material for: The interplay between temperature, Trypanosoma cruzi parasite load, and nutrition: Their effects on the development and life-cycle of the Chagas disease vector Rhodnius prolixus
Source: PLoS Negl Trop Dis. 2024 Feb 2;18(2):e0011937. doi: 10.1371/journal.pntd.0011937 (PMC10866482; doi:10.1371/journal.pntd.0011937)
Supplement: S1 GEE Models — (DOCX) [file pntd.0011937.s001.docx]

GEE Models

All formulas and results presented here were created and obtained using the *geepack* (1) and *gee* packages (2) in R (version 4.2.1)(3).

1. Differences in retention performance depending on infection, temperature and developmental stage:

GEE formula:

geeglm (formula = RetentionPerformance ~ DevelopmentalStage + Temperature * Infection,

family = gaussian, data = d, id = Data, corstr = "exchangeable")

Coefficients:

| Variable | Estimate | Std. Err | Wald | Pr(>\|W\|) |
| --- | --- | --- | --- | --- |
| Intercept | 0.56189 | 0.01014 | 3073.2 | <2e-16 *** |
| DevelopmentalStage | -0.06119 | 0.00178 | 1182.39 | <2e-16 *** |
| Temperature26 | 0.00151 | 0.00685 | 0.05 | 0.8258 |
| Temperature28 | 0.01732 | 0.00758 | 5.22 | 0.0224 * |
| Temperature30 | -0.01714 | 0.00683 | 6.3 | 0.0121 * |
| InfectionInfected | 0.01134 | 0.00797 | 2.03 | 0.1547 |
| Temperature26:Infection | -0.00199 | 0.00999 | 0.04 | 0.8422 |
| Temperature28:Infection | -0.03654 | 0.01203 | 9.23 | 0.0024 ** |
| Temperature30:Infection | -0.01346 | 0.01013 | 1.76 | 0.1842 |

Signification of codes: 0 “***” 0.001 “**” 0.01 “*” 0.05 “.”

Number of clusters: 173 Maximum cluster size: 4

QIC:

QIC QICU Quasi Lik CIC Params QICC

- 1. 19.225 -0.612 9.090 9 19.778

1. GEE model 2: differences in retention performance depending on infection, temperature and developmental stage without insects from the 28ºC treatment:

GEE formula:

geeglm (formula = RetentionPerformance ~ DevelopmentalStage + Temperature * Infection,

family = gaussian, data = dWithout28, id = Data, corstr = "exchangeable")

Coefficients:

| Variable | Estimate | Std. Err | Wald | Pr(>\|W\|) |
| --- | --- | --- | --- | --- |
| Intercept | 0.55492 | 0.01073 | 2672.44 | <2e-16 *** |
| DevelopmentalStage | -0.05962 | 0.00190 | 988.74 | <2e-16 *** |
| Temperature26 | 0.00159 | 0.00683 | 0.05 | 0.816 |
| Temperature30 | -0.01702 | 0.00682 | 6.22 | 0.013 * |
| InfectionInfected | 0.01129 | 0.00790 | 2.04 | 0.153 |
| Temperature26:Infection | -0.00189 | 0.00995 | 0.04 | 0.849 |
| Temperature30:Infection | -0.01331 | 0.01007 | 1.75 | 0.186 |

Signification of codes: 0 “***” 0.001 “**” 0.01 “*” 0.05 “.”

Number of clusters: 132 Maximum cluster size: 4

QIC:

QIC QICU Quasi Lik CIC Params QICC

13.89 14.88 -0.44 6.51 7 14.21

1. GEE model 3: differences in retention performance depending on Infection, developmental stage, and the quantity of blood ingested in the preceding instar (bloodingestedstd) in insects from the 28Cº treatment.

GEE formula:

geeglm (formula = RetentionPerformance ~ DevelopmentalStage + bloodingestedstd * Infection, family = gaussian, data = 28, id = Data, corstr = "exchangeable")

Coefficients:

| Variable | Estimate | Std. Err | Wald | Pr(>\|W\|) |
| --- | --- | --- | --- | --- |
| Intercept | 5.86e-01 | 3.23e-02 | 328.24 | <2e-16 *** |
| DevelopmentalStage | -5.96e-02 | 8.06e-03 | 54.66 | 1.4e-13 *** |
| InfectionInfected | -3.72e-02 | 1.30e-02 | 8.18 | 0.0042 ** |
| bloodingestedstd | -1.30e-04 | 6.34e-05 | 4.21 | 0.0401 * |
| Bloodingestedstd:Infection | 1.20e-04 | 6.47e-05 | 3.47 | 0.0623 . |

Signification of codes: 0 “***” 0.001 “**” 0.01 “*” 0.05 “.”

Number of clusters: 41 Maximum cluster size: 4

1. GEE model 4: differences in retention performance depending on Temperature and developmental stage comparing insects from the 26Cº group to insects from the 28Cº and 30Cº treatments.

GEE formula:

geeglm (formula = RetentionPerformance ~ DevelopmentalStage + Temperature, family = gaussian, data = dWithout24, id = Data, corstr = "exchangeable")

Coefficients:

| Variable | Estimate | Std. Err | Wald | Pr(>\|W\|) |
| --- | --- | --- | --- | --- |
| Intercept | 0.57758 | 0.00963 | 3599.18 | <2e-16 *** |
| DevelopmentalStage | -0.06337 | 0.00202 | 986.55 | <2e-16 *** |
| Temperature28 | -0.00135 | 0.00584 | 0.05 | 0.82 |
| Temperature30 | -0.02454 | 0.00441 | 30.97 | 2.6e-08 *** |

Signification of codes: 0 “***” 0.001 “**” 0.01 “*” 0.05 “.”

Number of clusters: 127 Maximum cluster size: 4

QIC:

QIC QICU Quasi Lik CIC Params QICC

9.22 8.90 -0.45 4.16 4 9.36

1. GEE model 5: differences in retention performance depending on Temperature and developmental stage comparing insects from the 28Cº group to insects from the 30Cº treatment.

GEE formula:

geeglm (formula = RetentionPerformance ~ DevelopmentalStage + Temperature, family = gaussian, data = dWithout24and26, id = Data, corstr = "exchangeable")

Coefficients:

| Variable | Estimate | Std. Err | Wald | Pr(>\|W\|) |
| --- | --- | --- | --- | --- |
| Intercept | 0.57242 | 0.01465 | 1526.0 | <2e-16 *** |
| DevelopmentalStage | -0.06253 | 0.00295 | 448.9 | <2e-16 *** |
| Temperature30 | -0.02303 | 0.00586 | 15.4 | 8.5e-05 *** |

Signification of codes: 0 “***” 0.001 “**” 0.01 “*” 0.05 “.”

Number of clusters: 81 Maximum cluster size: 4

QIC:

QIC QICU Quasi Lik CIC Params QICC

6.688 6.693 -0.346 2.997 3 6.835

1. GEE model 6: differences in parasite concentration in released urine depending on unfed weight, developmental stage and temperature in nymphs.

GEE formula:

geeglm (formula = ParasiteConcentration ~ DevelopmentalStage + Temperature * UnfedWeight, family = poisson, data = Nymphs, id = Data, corstr = "exchangeable")

Coefficients:

| Variable | Estimate | Std. Err | Wald | Pr(>\|W\|) |
| --- | --- | --- | --- | --- |
| Intercept | 4.00739 | 0.61886 | 41.93 | 9.5e-11 *** |
| DevelopmentalStage | 0.47582 | 0.19199 | 6.14 | 0.013 * |
| Temperature26 | -0.74963 | 0.31284 | 5.74 | 0.017 * |
| Temperature28 | 0.43111 | 0.33390 | 1.67 | 0.197 |
| Temperature30 | -0.11731 | 0.30486 | 0.15 | 0.700 |
| UnfedWeight | -0.00417 | 0.00828 | 0.25 | 0.615 |
| Temperature26:UnfedWeight | 0.03520 | 0.0691 | 25.99 | 3.4e-07 *** |
| Temperature28:UnfedWeight | 0.00531 | 0.00646 | 0.68 | 0.411 |
| Temperature30:UnfedWeight | 0.01957 | 0.00789 | 6.15 | 0.013 * |

Signification of codes: 0 “***” 0.001 “**” 0.01 “*” 0.05 “.”

Number of clusters: 75 Maximum cluster size: 3

QIC:

QIC QICU Quasi Lik CIC Params QICC

-8.75e+05 -8.75e+05 4.37e+05 6.71 9 -8.75e+05

1. GEE model 7: differences in parasite concentration in released urine comparing nymphs from the 24Cº group to all other temperature treatments.

GEE formula:

geeglm (formula = ParasiteConcentration ~ DevelopmentalStage + Temperature, family = poisson, data = Nymphs, id = Data, corstr = "exchangeable")

Coefficients:

| Variable | Estimate | Std. Err | Wald | Pr(>\|W\|) |
| --- | --- | --- | --- | --- |
| Intercept | 2.7061 | 0.4370 | 38.35 | 5.9e-10 *** |
| DevelopmentalStage | 0.7480 | 0.0928 | 64.90 | 7.8e-16 *** |
| Temperature26 | 0.4606 | 0.2139 | 4.64 | 0.031 * |
| Temperature28 | 0.6074 | 0.1967 | 9.53 | 0.002 ** |
| Temperature30 | 0.4989 | 0.2022 | 6.08 | 0.014 * |

Signification of codes: 0 “***” 0.001 “**” 0.01 “*” 0.05 “.”

Number of clusters: 75 Maximum cluster size: 3

QIC:

QIC QICU Quasi Lik CIC Params QICC

-8.69e+05 -8.69e+05 4.34e+05 4.83 5 -8.69e+05

1. GEE model 8: differences in parasite concentration in released urine depending on unfed weight and developmental stage in insects from the 26Cº treatment.

GEE formula:

geeglm (formula = ParasiteConcentration ~ DevelopmentalStage * UnfedWeight, family = poisson, data = Nymphs, id = Data, corstr = "exchangeable")

Coefficients:

| Variable | Estimate | Std. Err | Wald | Pr(>\|W\|) |
| --- | --- | --- | --- | --- |
| Intercept | -2.52180 | 1.62539 | 2.407 | 0.120781 |
| DevelopmentalStage | 1.48418 | 0.39038 | 14.454 | 0.000144 *** |
| UnfedWeight | 0.54693 | 0.09774 | 31.311 | 2.20e-08 *** |
| DevelopmentalStage: UnfedWeight | -0.10065 | 0.01900 | 28.046 | 1.18e-07 *** |

Signification of codes: 0 “***” 0.001 “**” 0.01 “*” 0.05 “.”

Number of clusters: 22 Maximum cluster size: 3

QIC:

QIC QICU Quasi Lik CIC Params QICC

-2.775e+05 -2.775e+05 1.387e+05 3.027 4 -2.775e+05

1. GEE model 9: differences in total parasite released comparing nymphs from the 24Cº group to all other temperature treatments.

GEE formula:

geeglm (formula = TotalParasite ~ DevelopmentalStage + Temperature, family = poisson, data = Nymphs, id = Data, corstr = "exchangeable")

Coefficients:

| Variable | Estimate | Std. Err | Wald | Pr(>\|W\|) |
| --- | --- | --- | --- | --- |
| Intercept | 4.676 | 0.591 | 62.65 | 2.4e-15 *** |
| DevelopmentalStage | 1.166 | 0.123 | 90.00 | < 2e-16 *** |
| Temperature26 | 0.452 | 0.289 | 2.45 | 0.118 |
| Temperature28 | 0.577 | 0.250 | 5.33 | 0.021 * |
| Temperature30 | 0.458 | 0.284 | 2.60 | 0.107 |

Signification of codes: 0 “***” 0.001 “**” 0.01 “*” 0.05 “.”

Number of clusters: 75 Maximum cluster size: 3

QIC:

QIC QICU Quasi Lik CIC Params QICC

-6.84e+07 -6.84e+07 3.42e+07 6.59 5 -6.84e+07

1. GEE model 10: differences in molting time depending on infection, temperature and developmental stage.

GEE formula:

geeglm (formula = MoltingTime ~ DevelopmentalStage + Temperature * Infection, family = Gamma, data = d, id = Data, corstr = "exchangeable")

Coefficients:

| Variable | Estimate | Std. Err | Wald | Pr(>\|W\|) |
| --- | --- | --- | --- | --- |
| Intercept | 9.53e-02 | 1.03e-03 | 8570.48 | <2e-16 *** |
| DevelopmentalStage | -1.18e-02 | 2.56e-04 | 2142.91 | <2e-16 *** |
| Temperature26 | 1.17e-02 | 6.93e-04 | 285.16 | <2e-16 *** |
| Temperature28 | 1.77e-02 | 1.22e-03 | 210.23 | <2e-16 *** |
| Temperature30 | 3.29e-02 | 1.44e-03 | 519.93 | <2e-16 *** |
| InfectionInfected | -7.76e-04 | 6.79e-04 | 1.31 | 0.253 |
| Temperature26:Infection | -2.49e-03 | 1.28e-03 | 3.76 | 0.053 . |
| Temperature28:Infection | 3.00e-03 | 1.96e-03 | 2.35 | 0.125 |
| Temperature30:Infection | -1.11e-05 | 1.83e-03 | 0.00 | 0.995 |

Signification of codes: 0 “***” 0.001 “**” 0.01 “*” 0.05 “.”

Number of clusters: 197 Maximum cluster size: 5

QIC:

QIC QICU Quasi Lik CIC Params QICC

1987.10 1994.86 -988.43 5.12 9 1987.38

1. GEE model 11: differences in molting time depending on infection and developmental stage in insects from the 26Cº treatment.

GEE formula:

geeglm (formula = MoltingTime ~ DevelopmentalStage + Infection, family = Gamma, data = 26, id = Data, corstr = "exchangeable")

Coefficients:

| Variable | Estimate | Std. Err | Wald | Pr(>\|W\|) |
| --- | --- | --- | --- | --- |
| Intercept | 0.109666 | 0.001890 | 3365.8 | <2e-16 *** |
| DevelopmentalStage | -0.012622 | 0.000531 | 564.0 | <2e-16 *** |
| InfectionInfected | -0.003244 | 0.001064 | 9.3 | 0.0023 ** |

Signification of codes: 0 “***” 0.001 “**” 0.01 “*” 0.05 “.”

Number of clusters: 51 Maximum cluster size: 5

QIC:

QIC QICU Quasi Lik CIC Params QICC

535.78 537.53 -265.77 2.12 3 535.97

1. GEE model 12: differences in urine production depending on infection, temperature, developmental stage and blood ingestion ratio:

GEE formula:

geeglm (formula = UrineProduction ~ DevelopmentalStage + Temperature * Infection * BloodIngestionRatio, family = poisson, data = Nymphs, id = Data, corstr = "exchangeable")

Coefficients:

| Variable | Estimate | Std. Err | Wald | Pr(>\|W\|) |
| --- | --- | --- | --- | --- |
| Intercept | -1.92291 | 0.25668 | 56.123 | 6.81e-16 *** |
| DevelopmentalStage | 1.03236 | 0.02816 | 1343.851 | <2e-16 *** |
| Temperature26 | 0.44718 | 0.32901 | 1.847 | 0.174 |
| Temperature28 | 0.26672 | 0.35517 | 0.564 | 0.453 |
| Temperature30 | -0.20517 | 0.30811 | 0.443 | 0.505 |
| InfectionInfected | 0.17279 | 0.31674 | 0.298 | 0.585 |
| BloodIngestionRatio | 0.14089 | 0.03324 | 17.962 | 2.25e-05 *** |
| Temperature26:Infection | -0.56901 | 0.44031 | 1.670 | 0.196 |
| Temperature28:Infection | -0.43522 | 0.51351 | 0.718 | 0.397 |
| Temperature30:Infection | -0.33376 | 0.41475 | 0.648 | 0.421 |
| Temperature26:BloodIngestionRatio | -0.07199 | 0.05324 | 1.828 | 0.176 |
| Temperature28:BloodIngestionRatio | -0.04159 | 0.05602 | 0.551 | 0.458 |
| Temperature30:BloodIngestionRatio | 0.03074 | 0.04537 | 0.459 | 0.498 |
| Infection:BloodIngestionRatio | -0.01510 | 0.05064 | 0.089 | 0.766 |
| Temperature26:Infection:BloodIngestionRatio | 0.06663 | 0.07180 | 0.861 | 0.353 |
| Temperature28:Infection:BloodIngestionRatio | 0.07079 | 0.08262 | 0.734 | 0.392 |
| Temperature30:Infection:BloodIngestionRatio | 0.06598 | 0.06288 | 1.101 | 0.294 |

Signification of codes: 0 “***” 0.001 “**” 0.01 “*” 0.05 “.”

Number of clusters: 185 Maximum cluster size: 5

QIC:

QIC QICU Quasi Lik CIC Params QICC

-65816.48 -65850.94 32942.47 34.23 17 -65815.38

Tools used:

1. Halekoh U, Højsgaard S, Yan J. The *R* Package **geepack** for Generalized Estimating Equations. J Stat Soft [Internet]. 2006 [cited 2023 Feb 13];15(2). Available from: http://www.jstatsoft.org/v15/i02/

2. Carey VJ. Carey VJ (2022). _gee: Generalized Estimation Equation Solver_. R package version 4.13-23, <https://CRAN.R-project.org/package=gee> [Internet]. 2022. Available from: https://CRAN.R-project.org/package=gee

3. R Core Team. R Core Team (2022). R: A language and environment for statistical computing. R Foundation for Statistical Computing, Vienna, Austria. URL https://www.R-project.org/. 2022.
