# Supplementary figures and images for: The interplay between temperature, Trypanosoma cruzi parasite load, and nutrition: Their effects on the development and life-cycle of the Chagas disease vector Rhodnius prolixus
Source: PLoS Negl Trop Dis. 2024 Feb 2;18(2):e0011937. doi: 10.1371/journal.pntd.0011937 (PMC10866482; doi:10.1371/journal.pntd.0011937)

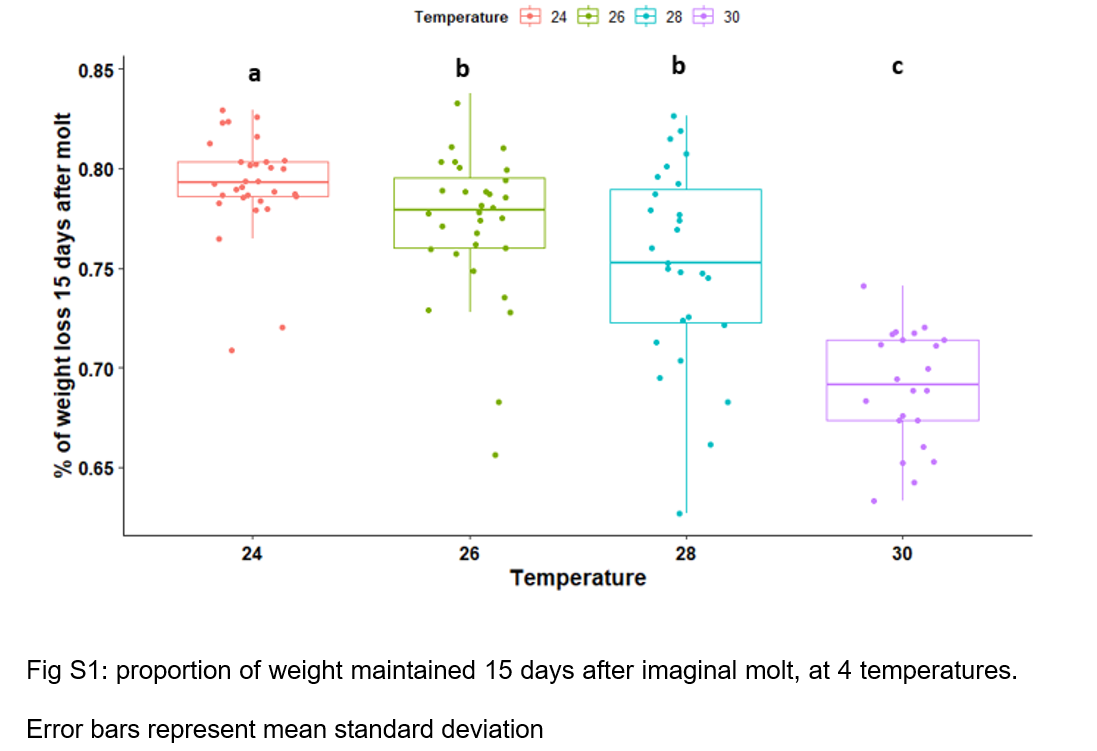

Supplement: S1 Fig — (TIF) [file pntd.0011937.s003.tif]

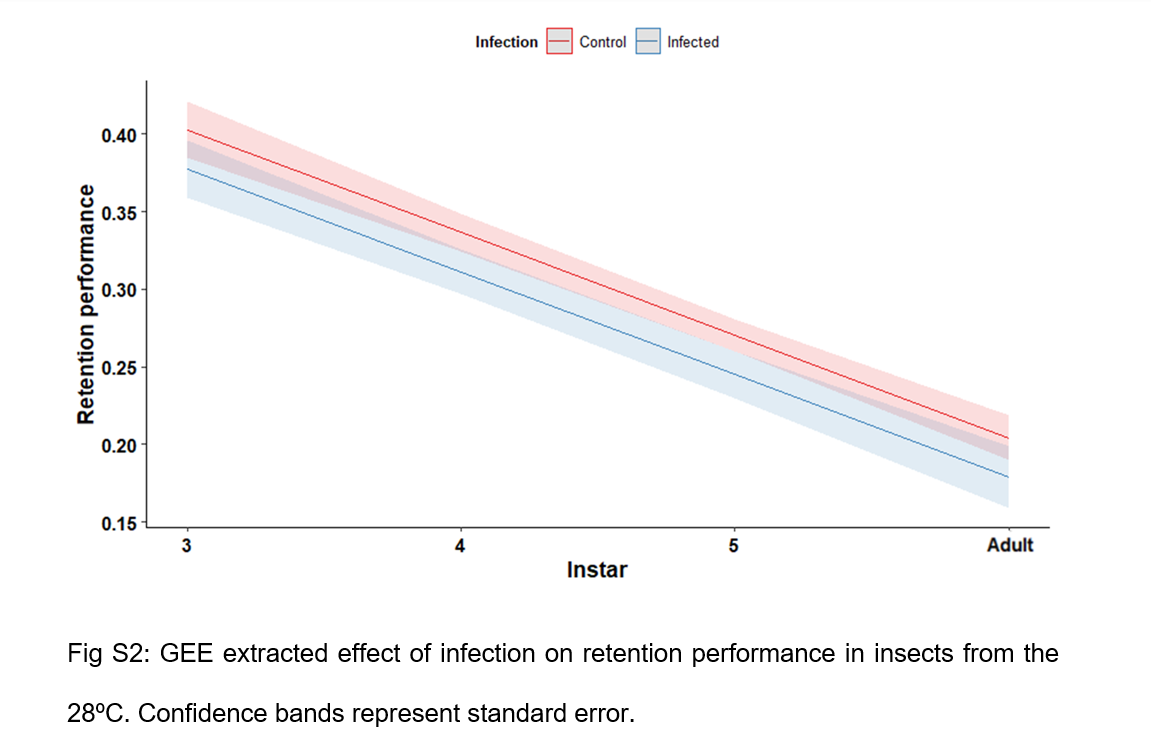

Supplement: S2 Fig — (TIF) [file pntd.0011937.s004.tif]

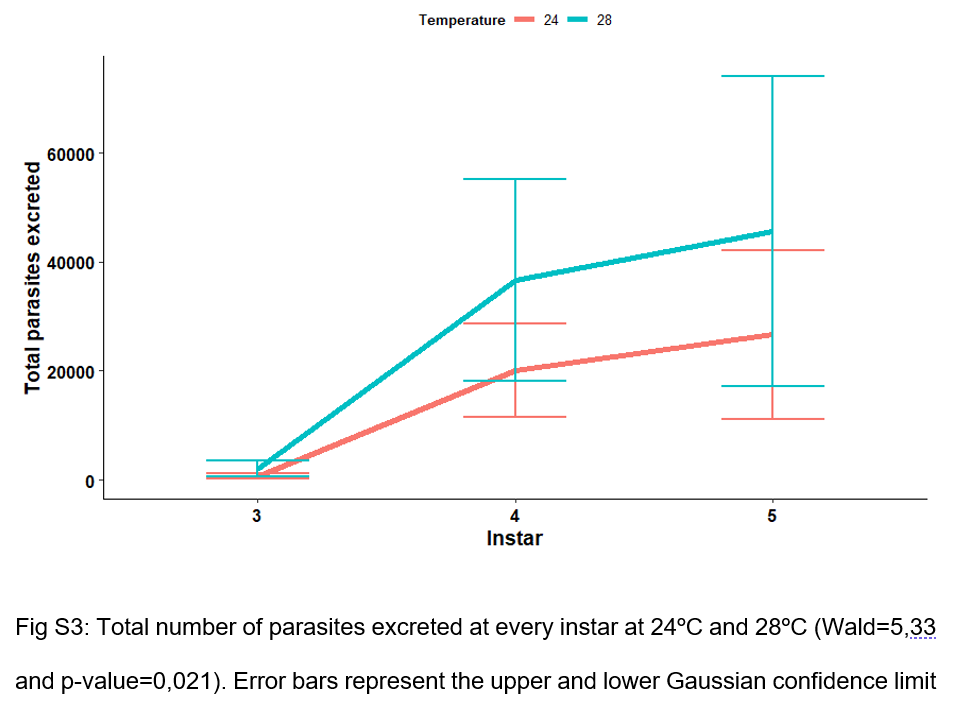

Supplement: S3 Fig — (TIF) [file pntd.0011937.s005.tif]

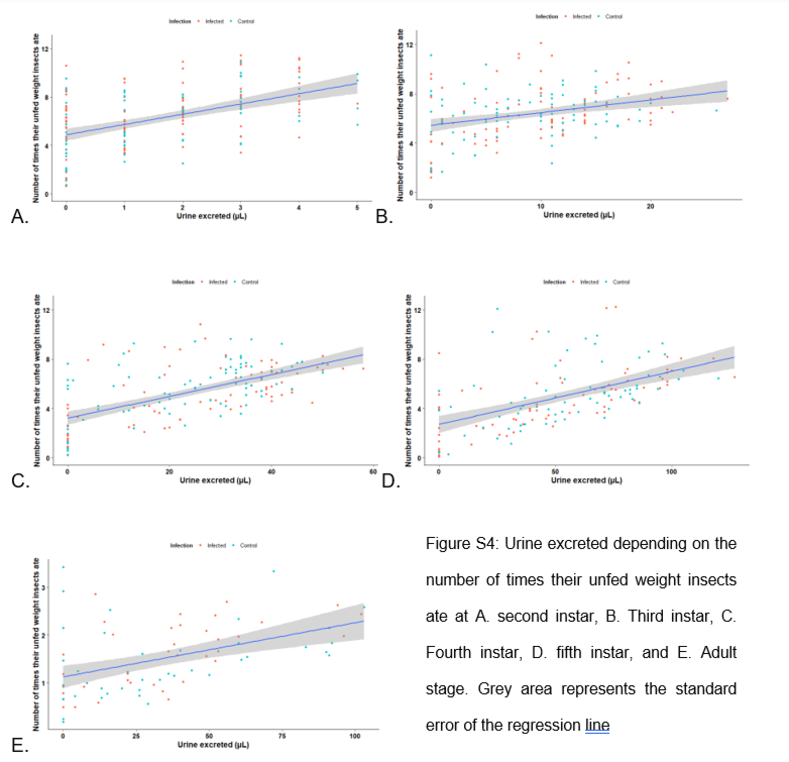

Supplement: S4 Fig — (TIF) [file pntd.0011937.s006.tif]
